# Extending the Linear Dynamic Range of Single Particle ICP-MS for the Quantification of Microplastics

**DOI:** 10.1021/acs.analchem.5c03552

**Published:** 2025-09-05

**Authors:** George C. Caceres, Monique E. Johnson, John L. Molloy, Sang Bok Lee, Antonio R. Montoro Bustos

**Affiliations:** † Chemical Sciences Division, Material Measurement Laboratory, 10833 National Institute of Standards and Technology, 100 Bureau Drive, Gaithersburg, Maryland 20899-1070, United States; ‡ Department of Chemistry and Biochemistry, 1068 University of Maryland, College Park, Maryland 20742, United States

## Abstract

In response to the growing concern of microplastics (1 μm to 5 mm) accumulation affecting human health, the development of analytical methods continues to be critical for the detection and characterization of microplastic particles. In this context, pursuing exceptional particle detection capability down to practical low levels and rapid analyses with high sample throughput makes single particle inductively coupled plasma mass spectrometry (spICP-MS) very attractive for microplastics analysis. Existing spICP-MS-based studies have routinely shown limitations in the accurate sizing and quantification of particle number concentration through targeting carbon content, with reported size limits of detection in the range of 0.62 to 1.8 μm and a substantial reduction in the transport of particles larger than 3 μm. In this work, the linear dynamic range of spICP-MS for the accurate quantification of polystyrene microparticles (PS MPs) via the monitoring of their carbon content (^13^C^+^) is extended to larger particle sizes (5 μm) through using a high efficiency sample introduction system with rigorous optimization of the ^13^C signal and operating at a lowered nebulizer gas flow to improve sample transport of larger particles to the plasma. Reliable quantification of particle number concentration (PNC), accepted as falling within 20% of expected particle stock concentrations, was achieved through a 20% lowered nebulizer gas flow for a full suite of commercial PS MPs ranging from 2 to 5 μm as well as a 2.2 and 4.8 μm PS MP contained within mixtures of the two materials, regardless of PNC ratio.

## Graphical abstract

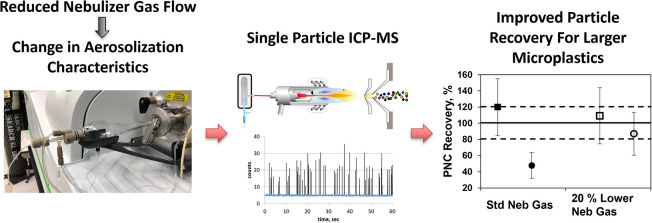

Microplastics are defined as plastic particles of nominal sizes between 1 μm and 5 mm.^1^ Interest in their study has grown exponentially over the past two decades, which comes as a response to their considerable ubiquity. The widespread nature of microplastics has raised great concern over their potential impact on human health and has led to greater efforts to improve our understanding of these impacts. The analytical techniques used to further our understanding of microplastics provide the core tools required to advance such research. Techniques such as scanning electron microscopy, transmission electron microscopy, dynamic light scattering, nanoparticle tracking analysis, pyrolysis gas chromatography–mass spectrometry, X-ray photoelectron spectroscopy, Raman spectroscopy, and Fourier transform infrared spectroscopy are all popular methods for the analysis of microplastics.^2,3^ While each analytical technique offers great advantages regarding key measurement aspects such as size determination and resolution, particle counting capabilities, polymer identification capabilities, sample integrity, ease of use, and cost, each also possesses shortfalls that can be addressed through supplementary techniques.^2,4^

Single particle inductively coupled plasma mass spectrometry (spICP-MS) is one such technique that has shown promise for use in the analysis of microplastics and is capable of offering specific benefits over those previously listed. The primary advantage of spICP-MS is particle number concentration (PNC) determination of particles at practically low concentrations (ranging from 10^3^ mL^–1^ to 10^5^ mL^–1^)^5^ and sizing capabilities down to 10 nm as observed in the analysis of metallic nanoparticles (NPs).^6^ However, analyzing microplastics using spICP-MS introduces several challenges. For example, ICP-MS determination of carbon in microplastics results in lower sensitivity compared to metallic NPs and high background levels arising from the interference of CO_2_ in the atmosphere and water. Both factors contribute to high carbon detection limits, in the range of mg L^–1^.^7–9^ Regarding the transport of plastic microparticles (MPs) into the plasma, challenges remain in achieving efficient nebulization, subsequent complete volatilization, and atomization of MPs within the ICP source, evidenced by the reported mass dependency of transport efficiency for NPs and MPs.^10^

Alternative avenues of analyzing microplastics via spICP-MS without carbon detection have been explored,^11–13^ however, the polymeric and carbon-based nature of microplastics make carbon detection the more straightforward and commonplace approach for quantitative analysis. For example, Bolea-Fernandez reported the use of ^13^C to investigate the quantification of 1 and 2.5 μm lanthanide-doped polystyrene (PS) MPs and Laborda et al. reported the application of ^13^C detection toward 2.2 and 4.8 μm PS MPs as well as those found in consumer products and river waters.^7–9^ Gonzalez de Vega reported a comparison of ^12^C and ^13^C detection in ultrapure water and seawater as well as strategies to improve lower detection limits of PS MPs down to 0.62 μm, using a triple quadrupole ICP-MS.^14^ Other efforts have examined complex mixtures of MPs, algae cells, and organic matter using an ICP-MS equipped with a time-of-flight mass analyzer (ICP-TOF-MS), assessed performance using conventional cyclonic spray chambers and concentric nebulizers, and implemented the use of a matrix independent calibration strategy using CO_2_ gas for the spICP-MS analysis of plastic MPs.^15–17^ While the number of studies examining the capabilities of spICP-MS for the analysis of microplastics continues to grow, when it comes to the measurement of MPs greater than 2 to 3 μm, lower nebulization efficiency, referring to the successful incorporation of particles in formed droplets, and particle transport to the ICP have been systematically reported across a variety of equipment setups, including other hyphenated techniques such as laser ablation ICP-MS.^7,10,15,16,18^ It should be noted, that in an attempt to improve particle transport of larger species, D. Gunther’s group adapted an ICP-MS with a downward pointing, vertical ICP for the purposes of providing a mass independent method of transport of particles or cells contained within large droplets.^19^ Expanding this ICP configuration to a TOF mass analyzer showed improvement in the transport efficiency of analyzed MPs sized between 3 to 20 μm. However, large uncertainties in the transport efficiency due to variability in the measurement of particle number concentration make accurate quantification using this ICP configuration difficult and less practical.^20^ Current research indicates that reliable quantification of PNC via spICP-MS with horizontal ICP configurations is limited to MPs in the size range of 1 to 3 μm, which is evidenced to be nebulized similarly to dissolved species and well-studied metallic NPs. Thus, PNC for larger MPs is underestimated, which is critical for the analysis of microplastics in real samples that exist in a broad size continuum.

Fundamental studies on the understanding of nebulization have shown that an increase of the nebulizer gas flow produces smaller and finer aerosol droplets which improves analyte transport.^21–23^ This phenomenon has so far been applicable to dissolved species and MPs smaller than 3 μm. As a result, the transport efficiency of larger MPs and intact bioparticles (cells, algae, bacteria, etc.) in the range of 1 to 30 μm has remained low until the advancement of nebulization technology and micronebulizers.^24^ Specific design considerations of micronebulizers such as smaller gas-exit cross sections, liquid cross sections, and a thinner wall of the liquid capillary produces more efficient liquid–gas interactions leading to finer aerosols with higher nebulization and transport efficiency relative to conventional pneumatic concentric nebulizers.^24,25^ Furthermore, critical dimensions and key properties like the gas-exit cross sectional area and nebulizer back pressure are directly influenced by the nebulizer gas flow rate.^25,26^ More recent investigations have shown that decreasing the nebulizer gas flow can improve the nebulization and transport of larger cells and, under certain conditions, offer comparable transport behavior to that of NPs.^27^ Based on this fundamental ICP theoretical framework, the effect of the nebulizer gas flow on the nebulization and transport of larger MPs via spICP-MS appears to be a reasonable avenue for investigation.

The goal of this study is to extend the linear dynamic range of spICP-MS quantification of MPs (beyond 3 μm) by improving nebulization efficiency of larger MPs when operating at a reduced nebulizer gas flow rate. Throughout this work, it is hypothesized that this would result in a similar nebulization efficiency and particle transport of larger PS MPs, dissolved carbon, and 30 nm AuNPs, leading to accurate PNC determination of PS MPs up to 5 μm.

## Experimental Section

### Chemicals

High purity water, prepared in-house by sub-boiling distillation using a conditioned quartz still with deionized water as feedstock, was used for dilution of all PS MPs, AuNPs, and dissolved ionic carbon and dissolved gold standards. NIST SRM 3121 (Gold (Au) Standard Solution Lot # 170531) and liquid chromatography-MS grade (99.8%, Sigma-Aldrich, St Louis, MO) methanol were used to prepare the dissolved gold and dissolved carbon calibration standards, respectively. While it is known that the use of methanol or other organic solvents alters the physical properties of the liquid solvent and affects nebulization and transport efficiency, the amount of methanol used in the prepared calibration solutions was low (<0.1% volume fraction) and is assumed to behave as an aqueous solution.^28,29^ The mass fractions and corresponding measured intensities of prepared calibration solutions used are listed in Tables S1 and S2. Very dilute aqueous suspensions of AuNPs and PS MPs, with approximate spherical shape, were analyzed in this study. LGC quality control material LGCQC5050 citrate stabilized, nominal diameter 30 nm colloidal AuNPs (LGC Ltd., Teddington, England), served as a calibration standard.^30^ Two polystyrene latex microspheres certified reference materials with nominal diameters of 2.2 and 4.8 μm and declared PNC of 2.58 × 10^10^ L^–1^ and 1.62 × 10^10^ L^–1^ (BCR-165 and BCR-166), obtained from the European Commission, Joint Research Centre (Geel, Belgium), functioned as method validation samples. Declared PNC for BCR-165 and BCR-166 were extracted from informational values in the final report based on particle counting investigations using a Coulter-type counter with a hydrodynamic focusing system on diluted test portions.^31^ NIST SRM 1690 polystyrene spheres of nominal diameter 1.0 μm^32^ and commercially available PS MPs of nominal diameters 1.0, 1.6, 2.0, 3.0, 4.0, and 5.0 μm (ThermoFisher Scientific, Waltham, MA) were used to assess of the linear dynamic range for the carbon-based analysis of microplastics by spICP-MS.

### Instrumentation

A NexION 350D quadrupole ICP-MS (PerkinElmer, Waltham, MA) was used for all spICP-MS measurements. Samples were introduced using a High Efficiency Sample Introduction System that includes a high efficiency MicroMist glass microflow concentric nebulizer (Glass Expansion, Melbourne, Australia) and a high-efficiency on-axis Lotis spray chamber (Glass Expansion, Melbourne, Australia) operated at room temperature. The sample flow rate was set to approximately 15 μL min^–1^ using the stock, built-in PerkinElmer NexION 350D peristaltic pump. While peristaltic pumps are known to fluctuate at such low flow rates, monitoring the flow rate over the course of 1 h prior to sample analysis and throughout the analysis period, the variance in the flow rate was observed to have a relative standard deviation no greater than 3%. Pump tubing was also changed frequently to prevent excessive stretching of the tubing. The ICP-MS was tuned daily for maximum ^115^In sensitivity while minimizing ^156^CeO/^140^Ce oxide levels (<2%). The ICP-MS was then tuned for maximum ^13^C and ^197^Au sensitivity through optimization of ion deflector voltages and rod voltage offsets. Such settings are then saved as separate conditions per analyte using the Syngistix software. An argon makeup gas, flowing through a microjet adapter designed to reduce aerosol droplet deposition on the spray chamber walls, was applied at a rate of 0.750 L min^–1^ and the nebulizer gas flow was optimized to be 0.360 L min^–1^. This optimized nebulizer gas flow will hereby be referred to as the “standard” nebulizer gas flow. Lowered nebulizer gas flow experiments were performed at a 20% lower rate than the standard nebulizer gas flow and was set to 0.288 L min^–1^. The makeup gas was left unchanged for the lowered nebulizer gas flow condition. The dwell time for all experiments was set to 100 μs and both ^13^C and ^197^Au sensitivities were recorded in time-resolved mode using the PerkinElmer Syngistix software’s nano module. Typical parameters for the carbon-based spICP-MS analysis of microplastics are listed in Table S3.

### Procedure

All PS MP and AuNP working suspensions were prepared fresh daily by gravimetric serial dilution of purchased stock suspensions to a target PNC of 2.5 × 10^7^ L^–1^ (equivalent to Au mass fraction of 3 pg g^–1^, and C mass fractions of 3 to 180 pg g^–1^ depending on the diameter). The target PNC was chosen to mitigate particle coincidence and to maintain sufficient particle flux. At each dilution stage, the sample was repeatedly inverted for 30 s, vortexed for 30 s, and bath-sonicated for 1 min. This aids in maintaining particle dispersity and minimizing potential particle agglomeration. Longer sonication times were not used to avoid excessive heating of suspensions.

### Data Processing and Calibration of spICP-MS

For each sample, a total of 3,000,000 data points were collected per 5 min analysis in units of counts and exported to Microsoft Excel for data processing. Data were consolidated using an in-house developed tool to transform the data collected at a dwell time of 100 μs into a data set resembling data collected at a dwell time of 1 ms for simpler processing. The data is transformed by combining ten sequential dwells to make a new 1 ms dwell period. Simultaneously, the data is parsed for particle events above an experimentally determined background intensity threshold to ensure particle events are not split between the newly formed dwell periods. The signal associated with the particles was then distinguished from the dissolved background signal using a 5σ criterion.^33^ The determination of PNC and particle size relies on the accurate calibration of the fraction of introduced sample that is transported into the plasma, termed “transport efficiency” (TE). In 2011, Pace and co-workers advanced two methods for the direct determination of TE in spICP-MS, the particle frequency method and the particle size method.^5^ Both methods rely on the use of a single NP reference material, which is currently scarce. In the particle frequency method (TEF), TE is measured as the ratio of the number of observed particle events to the number of particles introduced into the instrument (eq S1); TEF is thought to provide a measure of aerosol transport. In the particle size method (TES), TE is defined as the ratio of the measured intensity per mass of dissolved analyte to the observed intensity per mass of analyte for the NP reference material (eq S2); TES is thought to provide a measure of analyte transport. Here we adopt the protocol of using the TES measure of transport and the response factor (counts g^–1^) for dissolved carbon calibration standards to compute particle diameter of PS MPs, and the TEF measure of transport to compute PNC as outlined in eqs S3 and S4.^34–37^

### Uncertainty Analysis

A comprehensive evaluation of the expanded uncertainty associated with both particle size and PNC carbon-based spICP-MS measurements of microplastics has not been attempted and, therefore, remains a key aspect for method validation. As such, the uncertainty estimates were evaluated using type A and type B methods in accordance with the guidance provided in the “Guide to the Expression of Uncertainty in Measurement” (GUM).^38^ The standard uncertainties evaluated using type A and type B methods were then combined by taking the root sum of squares of all the individual uncertainty components and expanded at a 95% confidence interval (*U*95% C.I.) (*k* = 2). For particle size and PNC measurements, all recognized and evaluated sources of uncertainty, a description of how they were evaluated for each measurement component, and their relative contributions to the overall measurement uncertainty are listed in Tables S4 – S9.

## Results and Discussion

### Stability of PS MP Certified Reference Materials

To refine the protocol used throughout this work, the stability of BCR-165 and BCR-166 PS MPs were evaluated by spICP-MS over the course of a single experimental day (8 h) and throughout the following six consecutive days. To evaluate the stability over a single day, the number of particle events were observed every 12 min, which is the approximate measurement time for each sample (*n* = 1) (Figure S1). Immediately after the first measured sample, the observed number of particle events dropped to about 80% of the original particle events observed in the fresh suspension. For the nominal diameter 2.2 μm BCR-165, a plateau of around 71% of the originally observed particle events were exhibited after 60 min. For the nominal diameter 4.8 μm BCR-166, a plateau of around 53% of the originally observed particle events were seen after the same 60 min.

Throughout the course of 6 days, the observed number of particle events and intensity remained stable past the initial 60 min since preparation (*n* = 1) (Figure S2). However, the number of particle events recovered several days after the initial preparation of particle suspensions does not represent the full recovery capable of spICP-MS measurements of PS MPs. This presents a case that immediately after preparation of PS MP suspensions, the recoverable number of particle events begins to decrease due to potential factors such as particle aggregation and settling in aqueous media. This behavior is reported to be expected due to larger particle sizes relative to engineered NPs and densities greater than that of water for some microplastics.^39,40^ It is suspected that complete particle loss from this behavior was not observed due to the stabilizing effect of the diluted surfactant present in working suspensions. The effect of stirring on maintaining particle dispersity and homogeneity was also evaluated and observed to not influence the detected number of events (Figure S3). As such, it is recommended that PS MPs analyzed by spICP-MS be prepared fresh from stock each day, in addition to using particle dispersion techniques (e.g., sonication) known to aid in maintaining particle homogeneity of engineered NPs.^41,42^ Hence, the PS MPs analyzed in the remainder of this study were prepared fresh with sample analyses beginning within 10 min of preparation to ensure recovered particles are representative of a freshly diluted and dispersed particle suspension, not yet significantly affected by particle settling or aggregation.

### Effect of Nebulizer Gas Flow on Microparticle Transport Efficiency

As the nebulizer gas flow presents a promising avenue for improving particle transport of PS MPs, the effect of changing the nebulizer gas flow on the transport efficiency of PS MPs was investigated. BCR-165 and BCR-166 were analyzed in addition to LGCQC5050 as reference and calibration particles. The three particle materials were examined under two different nebulizer gas flow conditions, the standard nebulizer gas flow (0.360 L min^–1^) and the 20% lower nebulizer gas flow (0.288 L min^–1^). Other tests utilizing different nebulizer gas flow conditions were conducted and are discussed in the SI (Figure S4A) as well as repeatability testing of the optimum 20% lowered nebulizer gas flow (Figure S4B). Analyzing the TES or analyte transport at the standard nebulizer gas shows good agreement between all three particle materials from 30 nm to 5 μm. Although lowering the nebulizer gas flow by 20% did not statistically change the level of agreement of the two BCR materials relative to LGCQC5050, there was a noticeable increase in TES for BCR-166 between the standard and 20% lowered nebulizer gas flows (Figure 1A). This increase in the TES for BCR-166 is indicative of a lower particle response factor relative to the dissolved ionic response factor. A decrease in the particle response factor can result from a reduction in particle sensitivity per mass of analyte or a decrease in the linearity of the dynamic size range. A deviation from linearity in the dynamic size range was not observed at the reduced nebulizer gas flow condition (Figure S5) when compared to the same particles sized at the standard nebulizer gas flow (Figure S6). While linearity was not lost, a noticeable decrease in the slope of the linear regression when comparing these plots was observed, meaning there is a measurable reduction in proportionality of our measured particle sizes vs expected particle sizes when reducing the nebulizer gas flow. Between the standard and lower nebulizer gas flow conditions, particles smaller than 3 μm show nearly no change in the measured particle size whereas particles 3 μm and greater show a reduction in measured particle size of 4 to 8% as particle size increases from 3 to 5 μm, with the greatest difference being at the upper 5 μm end and the smallest difference at the 3 μm end. The slope of the linear regression between the two conditions for measured particle sizes reflects a similar change which can be explained by reduced sensitivity per mass of analyte at larger particle sizes. In fact, the normalized response factor for BCR-166 versus dissolved carbon decreased by 16% whereas normalized response factors for BCR-165 and LGCQC5050 increased by 9 to 10%, indicating a difference in analyte transport behavior for BCR-166 that is not reflected in the other analyzed particles. Thus, it is reasonable to conclude that lowering the nebulizer gas flow creates plasma conditions in which larger particles experience reduced sensitivity and higher TES as a result.

**Figure 1:**
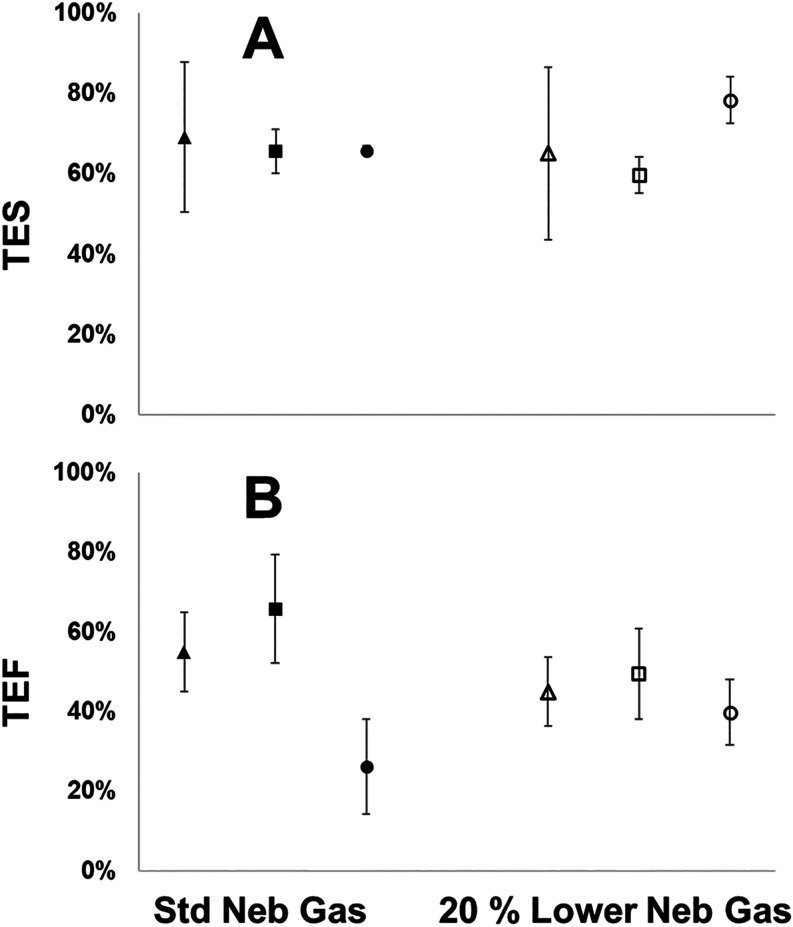
Transport efficiency computed via size method (TES) (A) and frequency method (TEF) (B) for LGCQC5050 30 nm AuNPs (▲/△), BCR-165 (■/□), and BCR-166 (●/○) at the standard nebulizer gas flow (0.36 L min^–1^) (▲, ■, ●) and reduced nebulizer gas flow (0.288 L min^–1^) (△, □, ○). Uncertainty shown represents a *U*95% based on *n* = 3 replicates.

To explore this idea further, it is known that the nebulizer gas flow affects plasma conditions in a way that alters the traversal behavior of ion clouds through the ICP. Critically, the nebulizer gas flow influences plasma temperatures and flow velocity into and throughout the plasma. Specifically, the gas flow can affect the position downstream of the plasma where the temperature rapidly rises, in which a higher nebulizer gas flow contributes to a rise in plasma temperature further downstream and a lower nebulizer gas flow contributes to a rise in temperature upstream of the plasma, or closer to the position of the torch injector.^43,44^ Such operating parameters create plasma conditions in which particles experience longer plasma residence times at hotter temperatures. Longer sample residence times in the plasma can lead to ion diffusion off-axis relative to the position of the sampling cone orifices that guide ions into the mass spectrometer. In addition, the sample aerosol exiting the torch injector undergoes changes to its flow velocity profile as the nebulizer gas flow is reduced to become wider and less defined the more the gas flow is reduced.^44,45^ Therefore, a widening of the central gas channel within the plasma channel in combination with a higher residence time could lead to diffusion loss of ions in other gas streams that do not enter the mass spectrometer;^45^ however, this behavior was only observed for BCR-166. With a particle mass an order of magnitude larger relative to BCR-165, the resulting ion cloud formed in the plasma for BCR-166 would also be much larger. This trend was also observed in the collected raw data. When changing nebulizer gas flow conditions from standard to 20% lower, LGCQC5050 and BCR-165 increased ion cloud lengths by an average of 100 to 200 μs while BCR-166 did not experience a proportional increase in its ion cloud length and instead was increased by an average of 300 μs (data not shown). The expanded ion clouds of these materials under the lowered nebulizer gas flow condition could encroach on the area of the central gas channel where it intersects with the auxiliary gas channel at the outer area of the plasma, before entering the mass spectrometer. This would result in a loss of ions and, therefore, sensitivity, primarily for larger particles. Despite the increase in TES, results still show agreement with the measured TES of LGCQC5050 at this condition and measured particle sizes still fall within the accepted criteria of 10% of the expected particle diameter. This would indicate that under the lowered nebulizer gas flow condition, there is complete volatilization, atomization, and transport of ions for LGCQC5050 and BCR-165 while providing an acceptable level of volatilization, atomization, and transport of ions for BCR-166 for accurate size measurements.

When analyzing the TEF or particle transport (Figure 1B), there is only complete nebulization and particle transport to the plasma for LGCQC5050 and BCR-165 at the standard nebulizer gas flow condition. The measured TEF for BCR-166 was found to be 26%, which represents a 53% lower TEF than that measured for LGCQC5050. This indicates that at these conditions, the larger BCR-166 are transported from the nebulizer into the plasma with less efficiency relative to the smaller LGCQC5050 and BCR-165 which, relative to each other, show good agreement between their measured TEF values of 55% and 65%, respectively. As mentioned above, there has been systematic reporting of lower particle transport for particles larger than 2 to 3 μm, likely due to issues with poor nebulization efficiency. Upon lowering the nebulizer gas flow, TEF for BCR-166 increases and lies in agreement with the TEF for both LGCQC5050 and BCR-165. This suggests that lowering the nebulizer gas flow creates the conditions necessary for a higher nebulization efficiency resulting in improved particle transport of PS MPs larger than 2 to 3 μm. The improvement in TEF for BCR-166 and its resulting agreement with the TEF of LGCQC5050 and BCR-165 presents a single set of operating conditions in which particles from 30 nm to 5 μm exhibit similar particle transport and analyte transport behavior. To the best of our knowledge, such conditions would enable, for the first time, accurate determination of PNC and size of these particles by spICP-MS. It should be noted that lowering the nebulizer gas flow reduced TEF measured for LGCQC5050 and BCR-165 relative to their respective TES values, where this was not the case at the standard nebulizer gas flow. Disagreement between TES and TEF has been reported previously^46^ and could arise due to a reduction in the size resolution and broadening of size distributions, effectively reducing the number of particles observed as the size range shifts below the practical detection limit. Nonetheless, this change in TEF did not diminish the ability to accurately measure PNC using frequency-based TE and is discussed further in context with the particle size distributions for both BCR PS MPs.

### Particle Size and Particle Size Distribution of PS MP Certified Reference Materials

Calibration of TES is necessary for the determination of the mean particle size and the particle size distributions. The agreement in TES between LGCQC5050, BCR-165, and BCR-166, used as method validation samples, enable the use of LGCQC5050 as the sole calibration standard for accurate sizing of PS MPs. It should be noted that value assignments accompanying commercially available NP and MP suspensions have been shown to be limited with respect to the number of particles analyzed and a more thorough characterization is needed.^47^ In this case, LGCQC5050 was verified in-house for particle size using high-resolution scanning electron microscopy for the analysis of over 5,000 individual particles.^37^

The measured mean particle sizes of BCR-165 and BCR-166 and their size distributions for standard and lowered nebulizer gas flows are shown in Figure 2. To minimize the sensitivity to outliers in these distributions, the Huber estimate of location^48^ was used as a measure of central tendency for particle diameter and the median absolute deviation (MAD) was selected to represent the spread of the particle size distributions. These representations of the data were selected as robust indicators for mean particle size and its associated variability. At the standard nebulizer gas flow, size distributions for both BCR materials (Figure 2A,B) are much broader than the size distributions originally reported for these materials by optical microscopy.^31^ Broadening of size distributions for micro- and nanoparticles by spICP-MS has been reported and discussed.^47,49^ The observed broadening effect is not unexpected due to the nature of the spICP-MS measurement process. The injection of particles off-axis produces particles following different paths within the plasma, partial ionization of particles, errors in ion counting, split particle events, and coincident particle events; all of which can give rise to a higher variability of detected particle intensities.^7,47,49,50^ The measured mean size and its full expanded uncertainty under the standard nebulizer gas flow for *n* = 3 subsamples of BCR-165 was determined to be 2.24 ± 0.21 μm which is 100.9% relative to the reported certified diameter and is in agreement with its associated expanded uncertainty.^31^ The mean size and full expanded uncertainty for *n* = 3 subsamples of BCR-166 was determined to be 4.81 ± 0.42 μm which is 99.7% relative to the reported certified diameter and is also in agreement with its associated expanded uncertainty.^31^ For both BCR materials, the relative *U*95% C.I. of the mean particle diameter represented 9.4 and 8.5% for BCR-165 and BCR-166, respectively, whereby the major source of uncertainty stems from the in-house value assigned mean particle diameter for LGCQC5050 (Tables S5 and S6).^37^ The theoretical size limit of detection (LOD_size_) for PS MPs analyzed in this study was computed as described by Laborda et al.^7^ and was determined to be 1.4 μm while the smallest MP practically observed was 1.8 μm. These values, while different, are still comparable to LOD_size_ determinations reported in the literature.^7,15,17,51^ As such, results obtained at the standard nebulizer gas flow serve as an appropriate baseline for discussion of results obtained at the reduced nebulizer gas flow.

**Figure 2:**
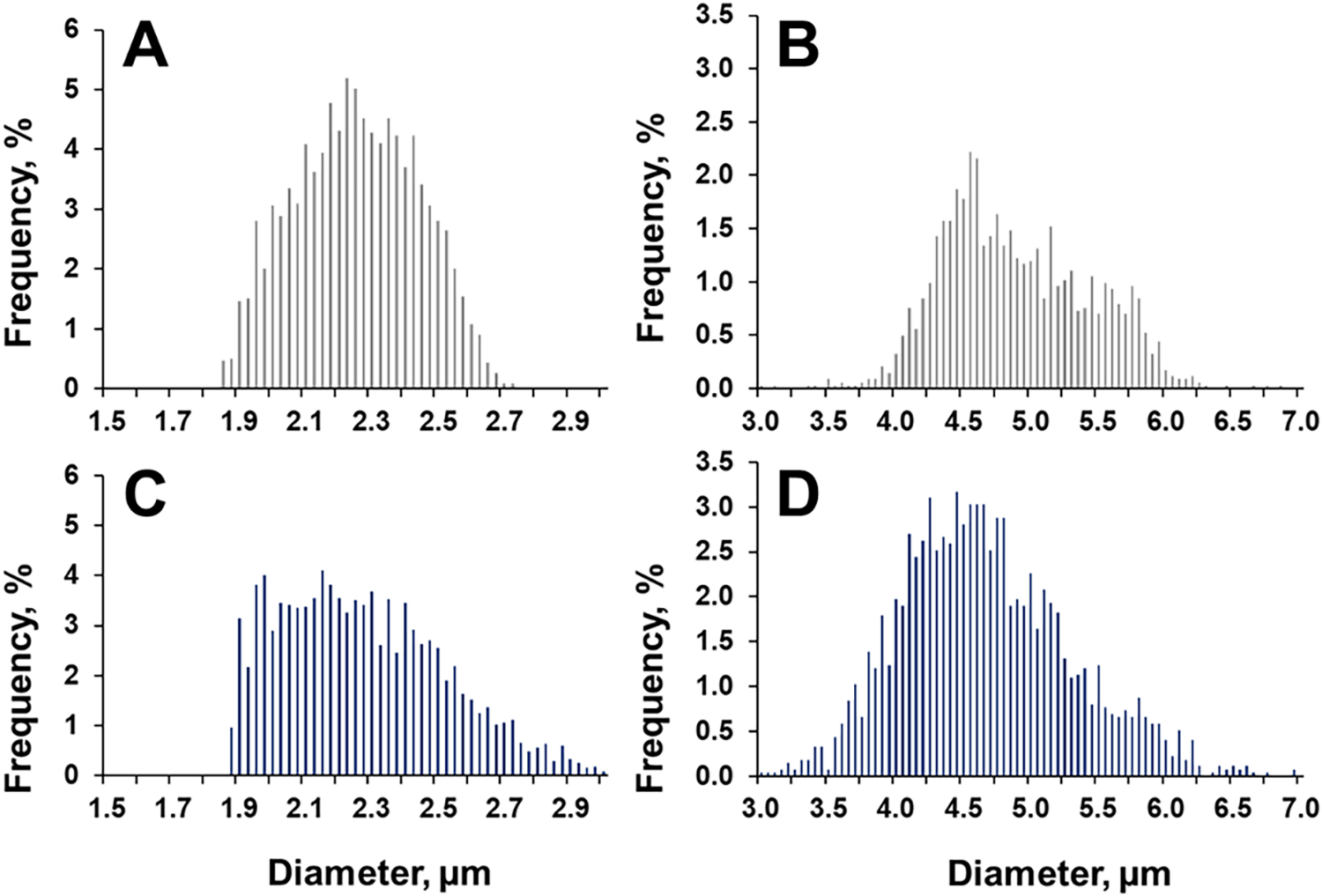
Particle size distribution histograms for PS MP BCR-165 (A and C) and PS MP BCR-166 (B and D) analyzed using the standard nebulizer gas flow of 0.36 L min^–1^ (Black) and reduced nebulizer gas flow of 0.288 L min^–1^ (Blue). A, B, C, and D were constructed from *n* = 3400, 1600, 4100, and 2700 analyzed particles, respectively.

Lowering the nebulizer gas flow induced changes to the size distributions of both BCR materials (Figure 2C,D). These changes were also reflected in the MAD where, under the standard nebulizer gas flow, BCR-165 and BCR-166 have a MAD of 0.14 and 0.39 μm, respectively, while at the lowered nebulizer gas flow, BCR-165 and BCR-166 have a MAD of 0.18 and 0.48 μm, respectively.

This increase in MAD is a more quantitative measure of the observed additional broadening in the size distribution histograms when lowering the nebulizer gas flow. As such, operating under the lowered nebulizer gas flow condition appears to enhance the broadening effects inherent to using an ICP as the ion source. BCR-165 exhibited a shift in the distribution that results from an inferior size resolution. This is an effect of reduced sensitivity from the lowered nebulizer gas flow that, while is reflected in a lower theoretical LOD_size_ of 1.2 μm, does not alter the practical detection limit of 1.8 μm. The observed shift in detected particle sizes toward the lower tail of the distribution indicates a higher abundance of smaller particle intensities and a general broadening of the intensity distribution. In fact, when lowering the nebulizer gas flow by 20%, the combined number of events of three replicates of BCR-165 measured in the lowest quartile of the size distribution increased by approximately 3% (data not shown). This contrasts with the trend observed in the overall number of events from these same three replicates which, approximately decreased by 10% (data not shown). It can then be hypothesized that a greater population of particles below the 1.8 μm threshold are also present. This would result in a particle size distribution that appears partially cut off, as is observed in Figure 2C. Loss of particles due to aggregation or particle coincidence was ruled out as the rate of particle coincidence for suspensions at 2.5 × 10^7^ L^–1^ under our experimental conditions, estimated by Poisson statistics,^44^ is negligible and there was no significant population of events detected per subsample (<5 events) in the expected size range for such events (3 to 6 μm). The reduction in particle events and resulting effects on the size distribution is also reflected in the mean PNC recovery for this material which will be discussed in greater detail in the following section. Although the shape of the particle size distribution for BCR-165 is shifted under these conditions, the ability to accurately measure the mean particle size has not diminished. The measured mean size under the lowered nebulizer gas flow for the BCR-165 was determined to be 2.24 ± 0.23 μm which is 100.7% relative to the reported certified diameter and lies in agreement with its associated expanded uncertainty.^31^ The size distribution for BCR-166 also displays slight broadening and a shift in the detected particle sizes due to the same phenomena affecting BCR-165 (Figure 2D). However, the Gaussian shape of the distribution is preserved as the measured intensities lie well above the experimental LOD_size_. The measured mean size under the lowered nebulizer gas flow for BCR-166 was determined to be 4.47 ± 0.44 μm which is approximately 7% lower than the mean size measured at the standard nebulizer gas flow. The lower mean size measured here is explained through the same reasoning made in the discussion of the measured TES for this material. Despite the lower particle size, the measured value remains within the accepted 10% range of the known size at 92.7% relative to the reported certified diameter.^31^

### Particle Number Concentration of PS MP Certified Reference Materials

The PNC for BCR-165 and BCR-166, used as method validation samples, were determined with traceability to the SI via LGCQC5050 which, is currently the only available particle material value assigned for PNC by a national metrology institute. Depicted in Figure 3, PNC for both BCR materials at the standard nebulizer gas flow were determined to be (3.09 ± 0.69) × 10^10^ L^–1^ and (0.78 ± 0.21) × 10^10^ L^–1^, respectively. These PNC values resulted in PNC recoveries of 119.8 ± 35.1% and 47.8 ± 15.9% for BCR-165 and BCR-166, respectively. The relative *U*95% C.I. represented 22.3 and 27.4% for BCR-165 and BCR-166, respectively, whereby the major source of uncertainty in the measurement stems from the value assigned PNC for LGCQC5050 (Tables S8 and S9).^30^

**Figure 3:**
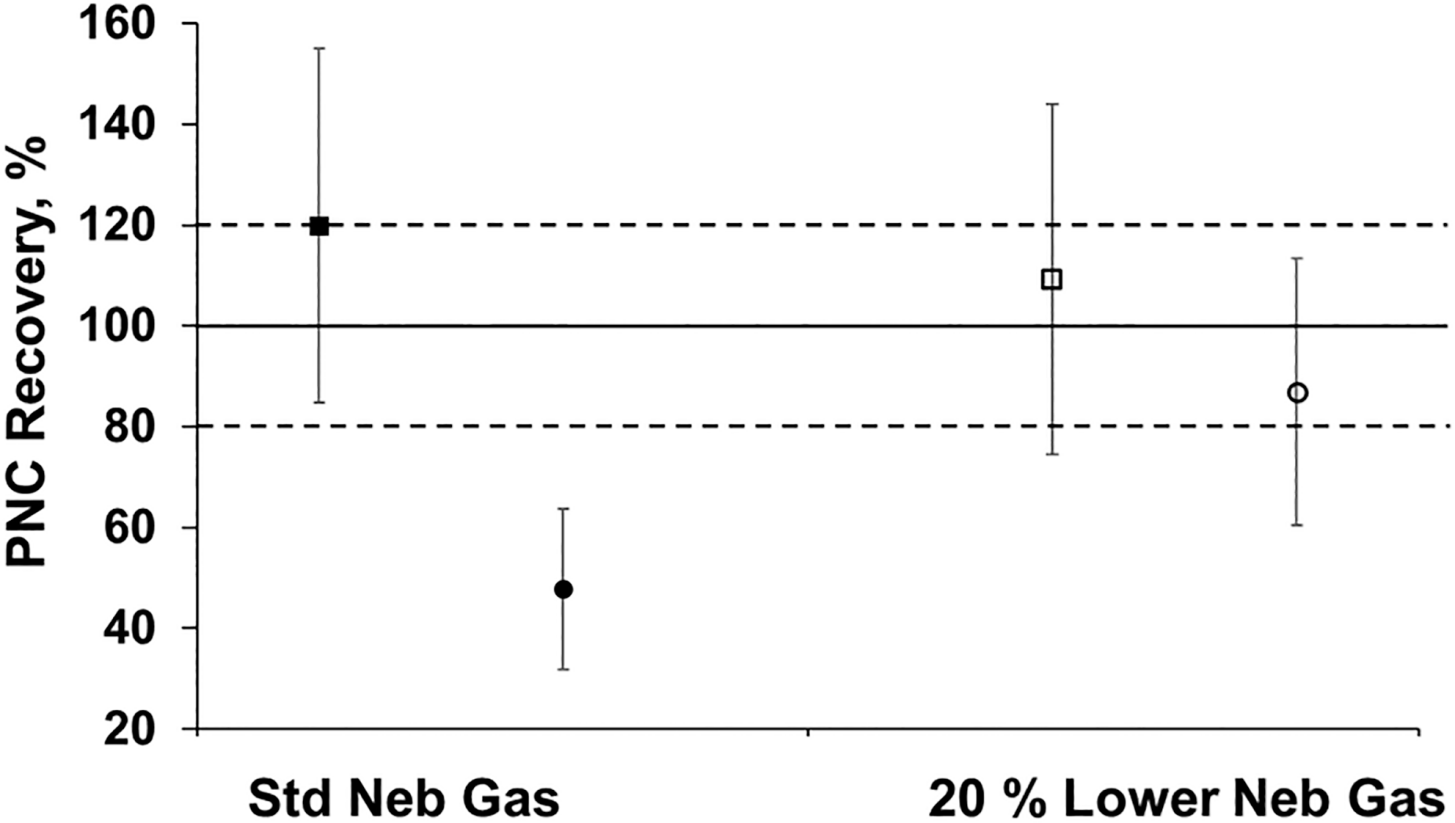
Particle number concentration recovery percent for PS MPs BCR-165 (■/□) and BCR-166 (●/○) analyzed under standard nebulizer gas flow conditions (0.36 L min^–1^) (■, ●) and reduced nebulizer gas flow conditions (0.288 L min^–1^) (□, ○). Uncertainty shown represents a *U*95% based on *n* = 3 replicate measurements.

BCR-165 shows good agreement with the expected stock PNC and is within the 20% tolerance range that is widely accepted for quantitative PNC measurements by spICP-MS. BCR-166 did not show acceptable agreement under these same conditions with a PNC recovery well outside the 20% tolerance range of the declared stock PNC for this material, supporting claims of different particle transport behavior for particles larger than 2 to 3 μm.^7,10,15,16,18^ Lowering the nebulizer gas flow rate by about 20%, the measured PNC was determined to be (2.82 ± 0.72) × 10^10^ L^–1^ and (1.41 ± 0.33) × 10^10^ L^–1^ for BCR-165 and BCR-166, respectively. At this condition, the measured PNC results in PNC recoveries of 109.2 ± 34.7% and 87.0 ± 26.4% for BCR-165 and BCR-166, respectively. Lowering the nebulizer gas flow improves particle recovery of BCR-166 in this case by approximately 40%, well beyond the error tolerance for the PNC measurement of this material at the standard nebulizer gas flow. This increase in PNC recovery is attributed to a change in aerosol production resulting from the reduced nebulizer gas flow. This new aerosolization behavior increases the efficiency of nebulization and transport of sample analytes into the plasma. Under these conditions, the particle transport of BCR-166 is now comparable to that of BCR-165 and LGCQC5050. As mentioned in the previous section, reducing the nebulizer gas flow impacts the intensities of detected particle events, particularly at the lower tail of the distribution of BCR-165. The 10% reduction in detected particle events is also reflected in the measured PNC as the PNC recovery for this material was reduced by 10% when changing conditions from standard to the lower nebulizer gas flow. However, despite a reduction in detected particle events for smaller MPs, we are still able to achieve quantitative PNC recovery for both BCR materials at a 20% lower nebulizer gas flow. The *U*95% C.I. for both BCR materials under this condition represented 25.5 and 23.4% for BCR-165 and BCR-166, respectively, where the major source of uncertainty was the value assigned PNC for LGCQC5050, similar to results at the standard nebulizer gas flow.

In addition to PNC, the limit of detection for the number of particles (LOD_PNC_) was evaluated under both standard and reduced nebulizer gas flow conditions over a period of five min. LOD_PNC_ was determined to be 60,000 and 80,000 L^–1^ at standard and 20% lowered nebulizer gas flow, respectively. LOD_PNC_ for both conditions are comparable to what has been reported previously via spICP-MS.^7,15^ While LOD_PNC_ can be improved with longer measurement times, a 5 min analysis time was chosen to balance detection of a sufficient number of events while keeping total sample run times reasonable (approximately 12 min).

### Particle Size and Particle Number Concentration of Commercial PS MPs Up to 5 μm

The two nebulizer gas flow conditions evaluated were then applied to a full suite of commercial PS MPs ranging from 0.9 to 5 μm, selected based on the lower size detection limit and the upper size limit for the working range of the ICP-MS pulse counting detector. For particle size determination, the standard nebulizer gas flow (Figure 4A) delivers accurate and reliable sizing for particles ranging from 1.6 to 5 μm. Measured diameters and their associated *U*95% C.I. for PS MPs within this size range are in good agreement (±10%) with manufacturer’s reported diameters, which are traceable to the SI through calibrations with NIST Standard Reference Materials. This result verifies previously reported linear behavior in the signal vs mass of PS MPs and indicates unbiased size determination within this range despite the poorer transport of MPs larger than 3 μm. Lowering the nebulizer gas flow yields similar linearity for particle size determination in the range of 1.6 to 5 μm, indicating that even under the changed plasma conditions resulting from the lowered nebulizer gas flow, there is still a similar level of volatilization and atomization of MPs and 30 nm AuNPs. Measured diameters and their associated *U*95% C.I. for PS MPs within this size range are also in good agreement with the manufacturer’s reported diameters (Figure 4B) at a 20% lowered nebulizer gas flow. While the determined size ratios of measured vs nominal particle diameters are, in general, lower for particles in the 1.6 to 5 μm range, the reduced sensitivity does not diminish the capability for accurate and reliable size determination of PS MPs. Signal vs mass of PS MPs linearity figures often represented in the literature were also recreated using data collected during this work (Figures S5 and S6). The LOD_size_ of our instrumental setup, reported above, hinders the accurate size determination of the 0.9 and 1 μm PS MPs, as seen in their much larger size ratio, indicating overestimation in the mean measured particle size of detected particle events (Figure 4A,B). PNC determination of the full suite of PS MPs showed greater differences between the two nebulizer gas flow conditions (Figure 4C,D). The standard nebulizer gas flow allows for quantitative PNC determination for only 2 and 3 μm PS MPs (Figure 4C), as expected based on the poorer particle transport of species larger than 3 μm described above and reported by spICP-MS.^7,15,16,18^ By lowering the nebulizer gas flow, we are able to extend the linear dynamic range of PNC quantification up to 5 μm (Figure 4D), which is at least 1 order of magnitude larger in carbon mass than previously achieved by spICP-MS. The PNC and associated *U*95% C.I. for PS MPs between 2 to 5 μm in diameter show good agreement (±20%) with estimated PNC stock values and show similar particle transport behavior across the entire size range in addition to 30 nm AuNPs. The remaining analyzed particles lie near or at the LOD_size_ so reliable quantification of PNC could not be achieved for these materials; however, this behavior is consistent with the literature. Particles with a nominal diameter larger than 5 μm were not tested because seemingly small increases in particle diameter equate to relatively large increases in particle mass which results in large increases in particle intensity. For example, an increase of 1 μm in nominal particle diameter from 5 to 6 μm is an increase of 1.2 times, however the resulting mass increase is approximately 1.7 times while the particle signal intensity is approximately 2 times larger under the reduced nebulizer gas flow condition. This would result in particle intensities that exceed the operating range of the pulse counting detector. Future work could examine the analysis of larger plastic MPs under optimized lower sensitivity conditions.

**Figure 4:**
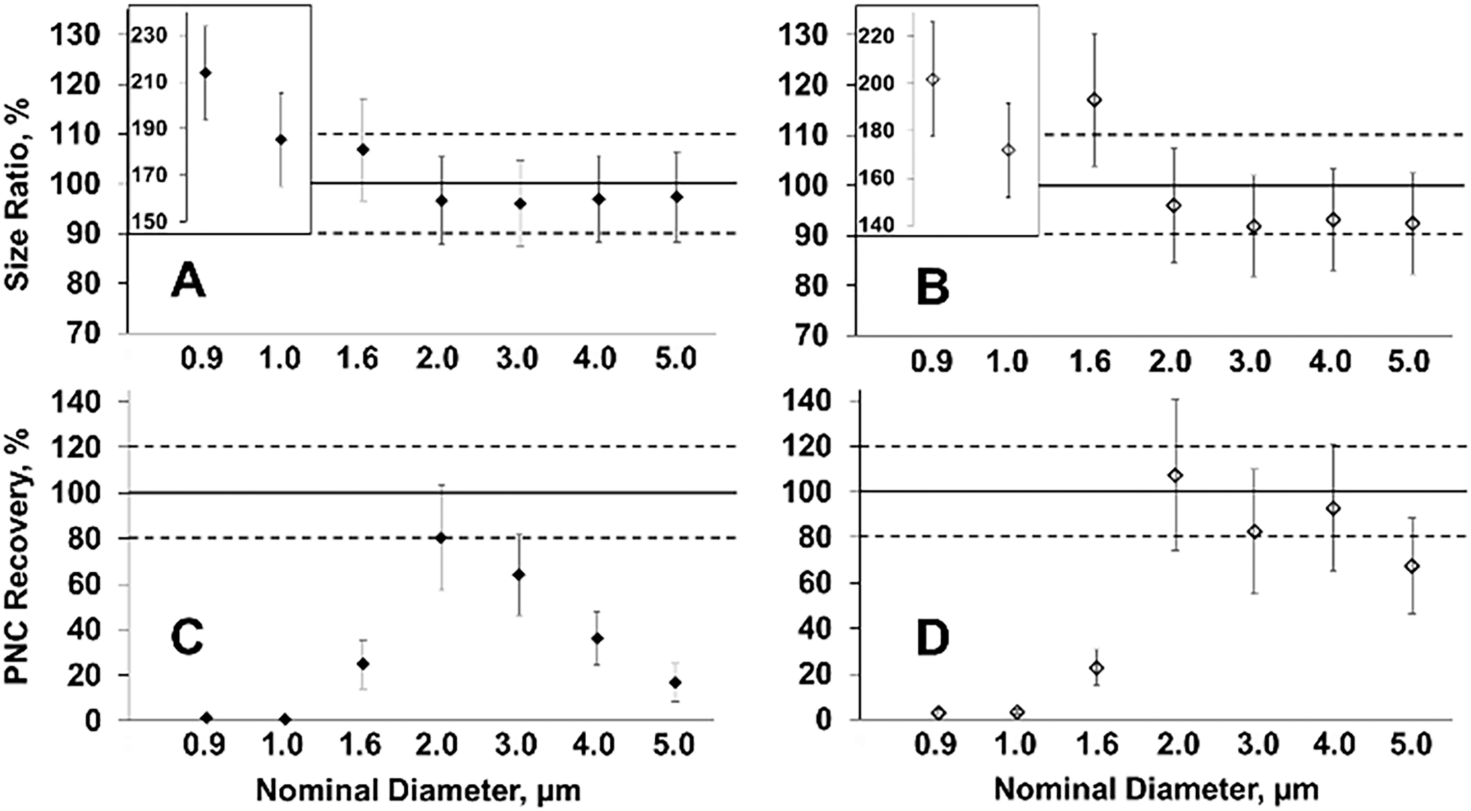
Size ratio between measured and expected particle diameters, expressed in percentage (A, B) and PNC recovery defined as the ratio of measured PNC to expected PNC, expressed in percentage (C, D) for a full suite of PS MPs with nominal sizes of 1.6, 2.0, 3.0, 4.0, and 5.0 μm analyzed under the standard nebulizer gas flow, 0.36 L min^–1^ (A, C) and the reduced nebulizer gas flow 0.288 L min^–1^ (B, D). Uncertainty shown represents a *U*95% based on *n* = 3 replicate measurements.

### Particle Size and Particle Number Concentration of PS MP Mixtures

Finally, the ability of our experimental setup was tested to resolve two different populations of PS MPs contained in a mixture. Three different PS MP mixtures were prepared using BCR-165 and BCR-166 at PNC ratios of 1:1, 5:1, and 1:5. Sizing results show the accurate determination of particle size for the two populations, regardless of the PNC ratios between the two materials and of the nebulizer gas flow used (Figure 5A,B). This indicates that with sufficient resolution between two different PS MP populations, sizing mixtures can be resolved with similar accuracy to individually prepared samples. Size distributions for BCR-165 and BCR-166 separated from these mixtures are shown in Figures S7 and S8. For PNC determination, it was hypothesized that reliable PNC quantification would be achieved for BCR-165 at both the standard and lowered nebulizer gas flow and for BCR-166 at only the lowered nebulizer gas flow. At the standard nebulizer gas flow, however, while PNC was quantifiable for BCR-165 for each mixture ratio, reliable quantification of PNC was unexpectedly observed for BCR-166 in samples with mixture ratios of 5:1 and 1:5 (Figure 5C). We suspect this behavior is a result of higher surfactant concentrations stemming from the significantly higher particle concentrations in these mixtures. Surfactant can alter solvent physical properties such as surface tension and viscosity which, in turn, could impact sample movement through the sample introduction system and behavior during aerosol formation.^19,52–54^ Altering solvent characteristics of MP suspensions could be a unique strategy to explore for PNC determination of more challenging samples in future work. Lowering the nebulizer gas flow, we observed reliable quantification of PNC across all mixture ratios for both BCR materials, similar to results obtained for individually prepared samples (Figure 5D). The 1:1 PNC ratio mixture notably results in an improvement in PNC recovery for BCR-166 when moving from the standard nebulizer gas flow condition to the lowered nebulizer gas flow which, exhibits particle recovery behavior similar to that of individually prepared BCR-166 and other commercial PS MPs with nominal sizes from 2 to 5 μm as part of the full suite shown in Figure 4. Through these results, the reliable quantification of PNC can be achieved for two different plastic MP species contained within the same suspension regardless of the PNC ratio when using the lowered nebulizer gas flow condition.

**Figure 5:**
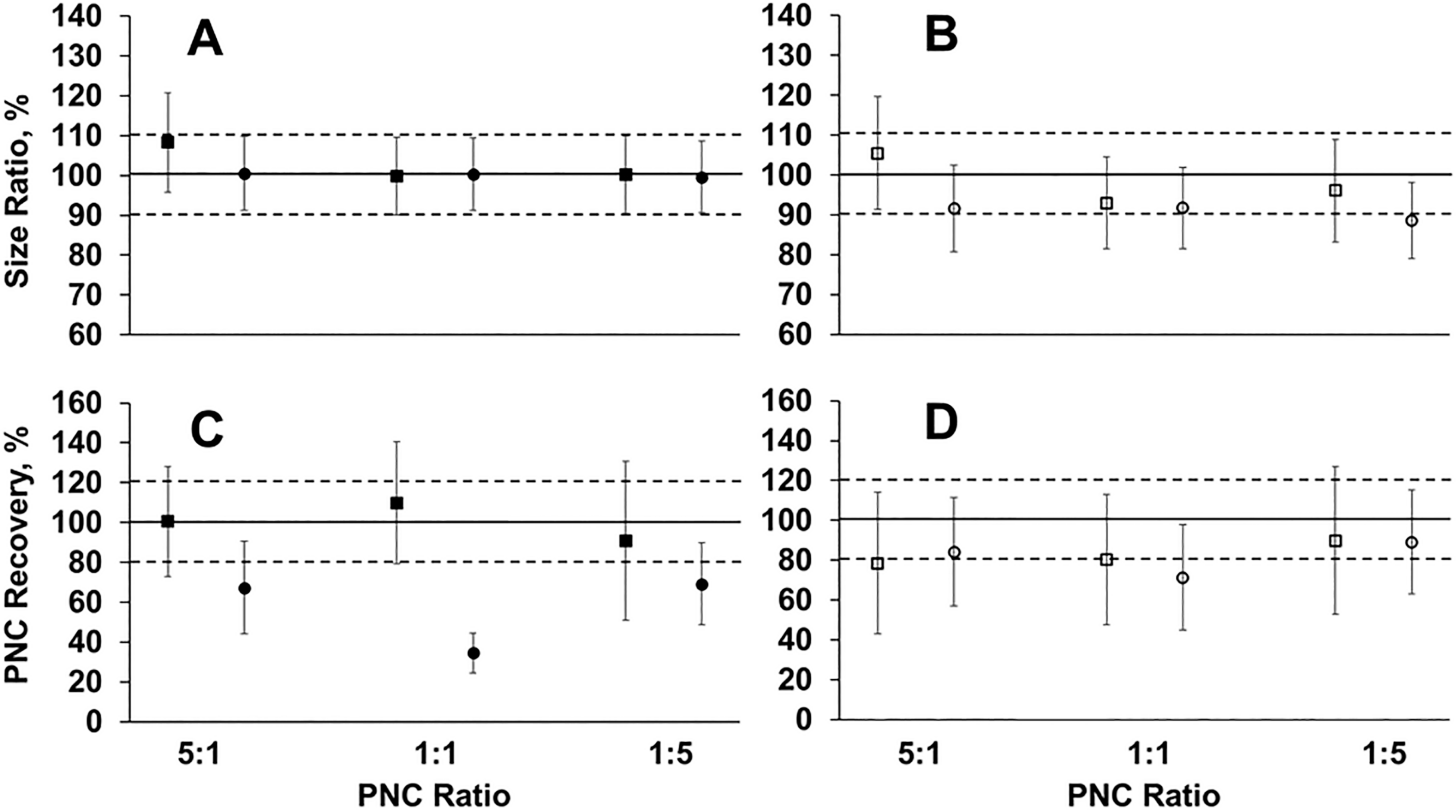
Size ratio between computed and expected particle diameters, expressed in percentage (A, B) and PNC recovery defined as the ratio of measured PNC to expected PNC, expressed in percentage (C, D) for PS MPs BCR-165 (■/□) and BCR-166 (●/○) present within mixtures of both materials prepared at PNC ratios of 1:1, 5:1, and 1:5. Mixtures were analyzed under the standard nebulizer gas flow, 0.36 L min^–1^ (A, C) and the reduced nebulizer gas flow 0.288 L min^–1^ (B, D). Uncertainty shown represents a *U*95% based on *n* = 3 replicate measurements.

## Conclusions

Current state of the art of spICP-MS for the analysis of MPs indicates that reliable PNC quantification is limited to particle diameters up to 2 to 3 μm. Beyond this size range, there is an observable divergence in particle transport behavior of larger MPs. Improvements in the design of sample introduction system components, intended for single cell research, have allowed the advancement of MPs analysis by spICP-MS. In addition, changes to the nebulizer gas flow have the capability to alter aerosol generation characteristics that affect sample transport behavior into the ICP. This study presents for the first time the development of an optimized set of operating conditions centered around a lowered nebulizer gas flow to achieve a similar particle transport behavior of larger plastic MPs to smaller plastic MPs and well-characterized AuNPs, effectively extending the linear dynamic range by nearly a factor of 2 for accurate quantification of PS MPs up to 5 μm by spICP-MS. This capability is an important milestone for spICP-MS analysis of MPs as this range is closer in size to some human cells like red and white blood cells. Plastic MPs can serve as calibrant materials or mock materials for the spICP-MS based analysis of other MPs or bioparticles, expanding capabilities to support further research of anthropogenic impacts on human health. However, additional work is needed to address falling transport efficiency of large cells and suitable microplastic counterparts, as a similar transport efficiency is exhibited across an extended size range for cells in comparison to plastic MPs.^55–57^ This could suggest a discrepancy between transport efficiency dependency on mass versus size.

Operating at a 20% lowered nebulizer gas flow resulted, for the first time, in comparable particle transport among three particle species ranging from 30 nm AuNPs to 5 μm PS MPs without introducing significant bias in analyte transport. Thus, accurate sizing and quantitative PNC recoveries were repeatably achieved for PS MPs of up to 5 μm, while only PS MPs up to 2 to 3 μm were reliably quantified at the standard nebulizer gas flow.

The robustness of the experimental setup for the accurate characterization of PS MPs was assessed through the analysis of employing a full suite of PS MPs from 0.9 to 5 μm. Linear behavior in the signal vs mass of PS MPs in the size range of 1.6 to 5 μm was consistently obtained for both tested nebulizer gas flow conditions. At the lowered nebulizer gas flow, accurate PNC quantification for the full suite of PS MPs confirmed results obtained for both BCR materials, while PNC recovery for only 2 and 3 μm PS MPs was reliably quantified at the standard nebulizer gas flow.

Furthermore, a comprehensive evaluation of the expanded uncertainty for the determination of the particle size of PS MPs revealed that the uncertainty associated with the determination of the mean particle size of the AuNP calibration standard was the dominant component in the uncertainty budget. Similarly, the uncertainty associated with the assigned value for PNC of the AuNP calibration standard was the main factor contributing to the uncertainty budget associated with the PNC quantification of PS MPs.

The ability of this system to resolve mixtures of PS MPs with different PNC ratios was also explored. Accurate sizing of both PS MP species was achieved within a single sample suspension across all tested ratios and both nebulizer gas flow conditions. PNC quantification exhibited the expected improvement at the lowered nebulizer gas flow across all ratios tested for the two PS MP species, however, at the standard nebulizer gas flow, only the 1:1 mixture showed poor PNC recovery of the 5 μm PS MP. It is plausible that the higher concentration of surfactant present in the 5:1 and 1:5 mixtures impacts the physical properties of the particle suspensions, leading to differences in aerosolization and impacting particle transport. Additional work is needed to systematically evaluate the effect of solvent characteristics on volatilization, atomization, and particle transport behavior of MPs during spICP-MS analysis.

## Supplementary Material



## Notes

Figures and additional information on the determination of transport efficiency, particle size, particle number concentration, testing of other nebulizer gas flow conditions, ICP-MS operating parameters for single particle analysis, elements of uncertainty determination, evaluation of practical stability of diluted BCR certified reference materials, and representation of particle size distributions andthe linear range of particle sizes for PS MPs examined throughout this work (PDF)
