# Supplementary material for: Extending the Linear Dynamic Range of Single Particle ICP-MS for the Quantification of Microplastics
Source: Anal Chem. 2025 Sep 5;97(36):19818–28. doi: 10.1021/acs.analchem.5c03552 (PMC12444753; doi:10.1021/acs.analchem.5c03552)
Supplement: Supplementary file 1 [file ac5c03552_si_001.pdf]

Supporting Information

Extending the Linear Dynamic Range of Single Particle ICP-MS for the Quantification of Microplastics

George C. Caceres[1,2], Monique E. Johnson,[1] John L. Molloy,[1] Sang Bok Lee,[2] Antonio R. Montoro Bustos[1]*

[1]Chemical Sciences Division, Material Measurement Laboratory, National Institute of Standards and Technology, 100 Bureau Drive, Gaithersburg, MD 20899-1070.

[2]Department of Chemistry and Biochemistry, University of Maryland, College Park, Maryland 20742.

Corresponding Author

* E-mail: antonio.montorobustos@nist.gov

ABSTRACT: Figures and additional information on the determination of transport efficiency, particle size, particle number concentration, testing of other nebulizer gas flow conditions, ICP-MS operating parameters for singe particle analysis, elements of uncertainty determination, evaluation of practical stability of diluted BCR certified reference materials, and representation of particle size distributions and the linear range of particle sizes for PS MPs examined throughout this work.

Table of Contents:

Transport efficiency determination…………………………………………………….S-4

Determination of particle diameter…………………………………………..……..S-5

Determination of particle number concentration……………………………………...S-5

Testing of other nebulizer gas flow conditions………………………………………...S-6

Table S-1. Prepared Au and C ionic calibration solutions employed at the standard nebulizer gas flow………………………………………………………………………..S-7

Table S-2. Prepared Au and C ionic calibration solutions employed at the 20 % lowered nebulizer gas flow………………………………………………………………………..S-8

Table S-3. Instrument operating and data acquisition parameters for spICP-MS ^{13}C analysis……………………………………………..S-9

Table S-4. Description of Components of Uncertainty for the spICP-MS Determination of the Particle Diameter of PS MPs...S-10

Table S-5. Individual Uncertainty Components for the spICP-MS Determination of the Particle Diameter of BCR-165 PS MP..……………..S-11

Table S-6. Individual Uncertainty Components for the spICP-MS Determination of the Particle Diameter of BCR-166 PS MP..……………..S-11

Table S-7. Description of Components of Uncertainty for the spICP-MS Determination of Number Concentration of PS MPs..S-12

Table S-8. Individual Uncertainty Components for the spICP-MS Determination of Number Concentration of BCR-165 PS MP………..S-13

Table S-9. Individual Uncertainty Components for the spICP-MS Determination of Number Concentration of BCR-166 PS MP………..S-13

Figure S-1. Stability of the number of measured particle events for BCR-165 and BCR-166 over 8 hours..S-14

Figure S-2. Stability of the number of measured particle events and particle intensity for BCR-165 and BCR-166 over 6 days……………………………………….................S-15

Figure S-3. Particle number concentration recovery of unstirred and stirred suspensions of BCR-165 and BCR-166……………………………………………………….…..S-16

Figure S-4. Particle number concentration recovery for suspensions of BCR-165 and BCR-166 under different nebulizer gas flow conditions and subsequent repeatability testing of optimal nebulizer gas flow conditions……………………………………...S-17

Figure S-5. Linearity of measured particle diameter vs. expected particle diameter for PS MPs at reduced nebulizer gas flow...S-18

Figure S-6. Linearity of measured particle diameter vs. expected particle diameter for PS MPs at standard nebulizer gas flow...S-19

Figure S-7. Size distributions of BCR-165 and BCR-166 contained in mixtures at the standard nebulizer gas flow…………………………………………………………S-20

Figure S-8. Size distributions of BCR-165 and BCR-166 contained in mixtures at the 20 % lowered nebulizer gas flow……………………………………………………….S-21

References……………………………………………………………………………S-22

Transport efficiency determination

The transport efficiency (TE), defined as the ratio of the amount of analyte entering the plasma to the amount aspirated, is a crucial parameter for the correct particle size and number determination in spICP-MS. Transport efficiency was determined daily using freshly diluted LGCQC5050 30 nm AuNP suspensions via the particle frequency method, which is denoted as *TEF*. *TEF* is computed as the ratio of the number of detected particles to the theoretical number of particles delivered to the ICP-MS by eq S1:

$$TEF = \frac{N_{NP\,RM}}{q_{liq} \times t_{aq} \times PNC_{RM}} \times DF_{RM} \quad \text{(S1)}$$

where *TEF* is the transport efficiency, $N_{NP\ RM}$ is the number of observed particle events for the calibration standard, q_{liq} (g min^{-1}) is the sample uptake rate, t_{aq} (min) is the time of acquisition, and PNC_{RM} (L^{-1}) is the certified value for PNC of LGCQC5050,[1] and DF_{RM} is the dilution factor of stock LGCQC5050 suspension.

Transport efficiency was also determined daily via the particle size method, which is denoted as *TES*. *TES* is computed as the ratio of the measured intensity of the dissolved analyte vs. the mass of the dissolved analyte to the observed signal intensity per mass of the analyte of the nanoparticle reference material by eq. S2:

$$TES = \frac{RF_{Ionic} \times d_{P\,RM}^{3} \times \pi \times \rho}{6 \times (I_{NP\,RM} - I_{diss\,RM})} \times 1E^{-12} \quad \text{(S2)}$$

where RF_{Ionic} (counts ng^{-1}) is the slope of the calibration curve formed by a plot of the intensity per mass of analyte for the working ionic standards, $d_{P\ RM}$ (nm) is the particle diameter of the particle reference material, ρ (g cm^{-1}) is the density of the particle reference material, $I_{NP\ RM}$ (Counts) is the intensity of measured particle events, and $I_{diss\ RM}$ (Counts) is the measured intensity of the dissolved background (note that $I_{diss\ RM} = I_{background} - I_{blk}$ where $I_{background}$ is all

signal not identified as a particle event and I_{blk} is the intensity for the water blank). Note that for TES for PS MPs, a correction factor of 0.9231, that is the carbon mass fraction in PS, needs to be applied in the numerator.

Determination of Particle Diameter

For the size determination of PS MP samples, *TES* was established from a response factor (Counts ng^{-1}) derived from the signal intensities measured for LGCQC5050, used as a calibration standard. Assuming that PS MPs are spherical, solid, have the density of the bulk analyte material, and that all samples exhibit a similar analyte transport efficiency in the plasma to LGCQC5050, the corresponding mass of each particle event was calculated via the measured *TES* and converted to particle diameter by eq S3:

$$d_p = \frac{6 \times (I_{NP} - I_{diss}) \times TES}{\pi \times \rho \times RF_{C\,Ionic} \times 0.9231 \times 1E^{-12}} \text{ (S3)}$$

where I_{NP} (counts) is the intensity of measured particle events, I_{diss} (counts) is the measured intensity of the dissolved background, ρ (g cm^{-1}) is the density of the particle, and $RF_{C\ Ionic}$ is the slope of the calibration curve formed from the intensity per mass of ^{13}C for the working carbon ionic standards, and 0.9231 is the carbon mass fraction in PS.

Determination of Particle Number Concentration

For the determination of PNC of PS MP samples, *TEF* was established from the number of detected events and certified PNC for LGCQC5050. LGCQC5050 is the only NP material certified for PNC by a national metrology institute, providing qunatifiable measurements of PNC traceable to the SI through its use as a calibration standard. Assuming all PS MPs exhibit similar particle transport efficiency to LGCQC5050, the measured number of particle events was converted to PNC using *TEF* by eq S4:

$$PNC = \frac{N_{NP}}{t_{analysis} \times q_{liq} \times TEF} \times DF \text{ (S4)}$$

where N_{NP} is the number of measured particle events, $t_{analysis}$ (min) is the duration of the measurement period, and q_{liq} (g min^{-1}) is the sample flow rate, and DF is the dilution factor of stock suspensions.

For purposes of computing the combined uncertainty of PNC of PS MP samples, eq S1 was substituted into eq S4 to form the final measurement equation for the spICP-MS measurement of PNC.

Testing of Other Nebulizer Gas Flow Conditions

Before arriving at the 20 % lowered nebulizer gas flow used throughout this work, other nebulizer gas flow conditions were tested. A noticeable increase in PNC recovery for BCR-166 was observed beginning with a 15 % reduction in the neb gas flow with the optimal improvement occuring at a 20 % reduction (Figure S4A). Further reduction of the nebulizer gas flow reduced sensitivity of LGCQC5050 to levels at or below background levels, making accurate quantification of PNC using these settings impossible. Additional experimentation to ensure repeatability of the 20 % lower nebulizer gas flow was conducted and presented in figure S4B. Testing conducted on a different day showed a consistent improvement in the PNC recovery for BCR-166 across two independent replicates. It is believed that these results in addition to those presented in figures 3, 4, and 5 show consistency in the improvement of PNC recovery under the applied conditions.

TABLES

Table S-1. Prepared Au and C ionic calibration solutions employed at the standard nebulizer gas flow

Gold (Au) from SRM 3121		Carbon (C) from methanol	
**Mass Fraction (ng/g)**	**Mean Intensity (Counts)**	**Mass Fraction (µg/g)**	**Mean Intensity (Counts)**
**0.00**	**0.77**	**0.00**	**34.02**
**0.91**	**14.44**	**28.94**	**164.85**
**4.46**	**88.88**	**54.70**	**276.71**
**17.58**	**347.00**	**132.97**	**628.49**
**47.06**	**920.83**	**272.66**	**1242.92**

Table S-2. Prepared Au and C ionic calibration solutions employed at the 20 % lowered nebulizer gas flow

Gold (Au) from SRM 3121		Carbon (C) from methanol	
**Mass Fraction (ng/g)**	**Mean Intensity (Counts)**	**Mass Fraction (µg/g)**	**Mean Intensity (Counts)**
**0.00**	**0.02**	**0.00**	**16.42**
**0.80**	**7.17**	**26.72**	**72.94**
**4.28**	**39.35**	**52.73**	**128.04**
**17.70**	**161.76**	**127.84**	**287.80**
**43.84**	**388.04**	**250.99**	**551.56**

Table S-3. Instrument operating and data acquisition parameters for spICP-MS ^{13}C analysis

Instrument	NexION 350D quadrupole ICP-MS
Sample introduction	Glass Expansion High Efficiency Sample Introduction System that includes a high-efficiency MicroMist^{TM} glass micro-flow concentric nebulizer and a high-efficiency on-axis Lotis^{TM} spray chamber operated at room temperature.
Type of Cones	Pt sampler, Pt skimmer and Al hyperskimmer
RF power (W)	1400
Plasma Ar gas flow (L min^{-1})	18.0
Auxiliary Ar gas flow (L min^{-1})	1.2
Nebulizer Ar gas flow (L min^{-1})	0.360 (standard), 0.288 (20 % lower)
Make up Ar gas flow (L min^{-1})	0.750
Sampling depth (mm)	11
Deflector Voltage (V)	-16 for ^{13}C, – 11 for ^{197}Au
Nominal sample flow rate (mL min^{-1})	0.015 (2-3 % RSD)
Dwell time (μs)	100
Acquisition time (s)	300

Table S-4. Description of Components of Uncertainty for the spICP-MS Determination of the Particle Diameter of PS MPs.

Component	Description	Type
u(TES)	Standard uncertainty of the measured size transport efficiency evaluated via Kragten spreadsheet described previously.[2, 3]	A
$u(I_{NP\ Sample})$	Standard uncertainty of the measured intensity of PS MPs, estimated as the maximum standard error of *n* = 3 replicates added in quadrature to the estimated standard error for the deadtime correction.	A
$u(I_{diss\ \mathrm{Sample}})$	Standard uncertainty of the measured intensity of the dissolved ^{13}C background, estimated as the maximum standard error of *n* = 3 replicate measurements.	A
$u(RF_{ionic})$	Standard uncertainty of the slope of the calibration curve formed by a plot of the intensity per mass of analyte for ^{13}C working ionic standards.	A
$u(\rho_{PS})$	Estimated standard uncertainty for the density of PS based on the variance of values reported in the literature.[4-9]	B
u(Rep)	Repeatability of samples represented by the relative standard error of the results for *n* = 3 subsamples.	A

Table S-5. Individual Uncertainty Components for the spICP-MS Determination of the Particle Diameter of BCR-165 PS MP.

Component	Typical Value (x_i)	$u(x_i)$	units	c_i	$c_i u_i$	ν	Rel.
$u(TES)$	0.6509	0.1058	--	1.18	0.125	122.33	96.2 %[a]
$u(I_{NP\ Sample})$	87.3	2.1	counts	$1.05x10^{-2}$	$5.00x10^{-4}$	23.55	3.1 %
$u(I_{diss\ \mathrm{Sample}})$	14.7	0.8	counts	$-1.05x10^{-2}$	$-8.74x10^{-3}$	3	0.5 %
$u(RF_{ionic})$	7763	10.8	counts ng^{-1}	$-9.83x10^{-5}$	$-1.06x10^{-3}$	3	0.0 %
$u(\rho_{PS})$	1.05	0.002	g cm^{-1}	-0.726	$-1.48x10^{-3}$	60	0.0 %
u(Rep)	1.000	0.007	--	0.763	$5.40x10^{-3}$	3	0.2 %
**Combined Type A**					**0.127**		
**Combined Type B**					**$1.48x10^{-3}$**		
**Combined standard uncertainty (u_c)**					**0.127**		

[a]While TES comprises 96.2 % of the overall measurement uncertainty for the measurement of particle diameter of BCR-165, the in-house value assigned mean particle diameter of LGCQC5050 represents 84.2 % and 61.8 % of the overall measurement uncertainty for the measurement of TES under standard and lowered nebulizer gas flow conditions, respectively.

Table S-6. Individual Uncertainty Components for the spICP-MS Determination of the Particle Diameter of BCR-166 PS MP.

Component	Typical Value (x_i)	$u(x_i)$	units	c_i	$c_i u_i$	ν	Rel.
$u(TES)$	0.6509	0.1058	--	2.33	0.236	122.79	98.1 %[a]
$u(I_{NP\ Sample})$	579.4	11.8	counts	$2.68x10^{-3}$	$3.15x10^{-2}$	23.55	1.7 %
$u(I_{diss\ \mathrm{Sample}})$	14.7	0.8	counts	$-2.68x10^{-3}$	$-1.16x10^{-3}$	3	0.0 %
$u(RF_{ionic})$	7763	10.8	counts ng^{-1}	$-1.95x10^{-4}$	$-2.09x10^{-3}$	3	0.0 %
$u(\rho_{PS})$	1.05	0.002	g cm^{-1}	-0.144	$-2.94x10^{-3}$	60	0.0 %
u(Rep)	1.000	0.006	--	0.151	$8.98x10^{-3}$	3	0.1 %
**Combined Type A**					**0.238**		
**Combined Type B**					**$2.94x10^{-3}$**		
**Combined standard uncertainty (u_c)**					**0.238**		

[a]While TES comprises 98.1 % of the overall measurement uncertainty for the measurement of particle diameter of BCR-166, the in-house value assigned mean particle diameter of LGCQC5050 represents 84.2 % and 61.8 % of the overall measurement uncertainty for the measurement of TES under standard and lowered nebulizer gas flow conditions, respectively.

Table S-7. Description of Components of Uncertainty for the spICP-MS Determination of the Number Concentration of PS MPs.

Component	Description	Type
$u(n_{\text{NP events Samp}})$	Standard uncertainty of the measured number of particle events, estimated as the maximum standard error of n = 3 5 min measurements for the samples.	A
$u(t_{\text{analysis NP RM}})$	Estimated standard uncertainty of the 5 min measurement period for the measurement of the NP RM calibration standard.	B
$u(q_{\text{liq NP RM}})$	Standard uncertainty of the sample flow, estimated as the standard error of n = 11, 15 min measurements of the mass of sample introduced into the instrument per unit of time for the spICP-MS analysis of NP RM calibration standard.	A
$u(n_{\text{NP events NP RM}})$	Standard uncertainty of the measured number of NP events, estimated as the maximum standard error of n = 3, 5 min measurements for the NP RM calibration standard.	A
$u(t_{\text{analysis Samp}})$	Estimated standard uncertainty of the 5 min measurement period for the measurement of the PS MPs samples.	B
$u(q_{\text{liq Samp}})$	Standard uncertainty of the sample flow, estimated as the standard error of n = 11, 15 min measurements of the mass of sample introduced into the instrument per unit of time for the spICP-MS analysis of the PS MPs samples.	A
$u(NC_{\text{NP RM}})$	Standard uncertainty reported for the assessed value of PNC of the stock suspension of the NP RM standard used for calibration.	B
$u(DF_{\text{NP RM}})$	Standard uncertainty of the gravimetric NP RM dilution factor. This was derived by combining in quadrature the estimated relative uncertainty for each mass measurement of the two-step dilution scheme. An uncertainty of magnitude ±0.00030 g in each measured mass on the 5-place analytical balance was assumed. This interval was divided by 2 to derive the half-width of the interval.	B
$u(DF_{\text{Samp}})$	Standard uncertainty of the gravimetric PS MP dilution factor. This was derived by combining in quadrature the estimated relative uncertainty for each mass measurement of the one-step dilution scheme. An uncertainty of magnitude ±0.00030 g in each measured mass on the 5-place analytical balance was assumed. This interval was divided by 2 to derive the half-width of the interval.	B
u(Rep)	Repeatability of samples represented by the relative standard error of the results for n = 3 subsamples.	A

Table S-8. Individual Uncertainty Components for the spICP-MS Determination of Number Concentration of BCR-165 PS MP.

Component	Typical Value (x_i)	$u(x_i)$	units	c_i	c_iu_i	ν	Rel.
$u(n_{\text{NP events Samp}})$	1006	31	particles	2.26x10^{4}	6.88x10^{5}	3	5.8 %
$u(t_{\text{analysis NP RM}})$	5.00	0.05	min	4.54x10^{6}	2.27x10^{5}	60	0.6 %
$u(q_{\text{liq NP RM}})$	0.015	5.6x10^{-4}	g min^{-1}	1.47x10^{9}	8.16x10^{5}	10	8.1 %
$u(n_{\text{NP events NP RM}})$	877	4.3	particles	-2.57x10^{4}	-1.12x10^{5}	2	0.2 %
$u(t_{\text{analysis Samp}})$	5.00	0.05	min	-4.49x10^{6}	-2.25x10^{5}	60	0.6 %
$u(q_{\text{liq Samp}})$	0.015	5.6x10^{-4}	g min^{-1}	-1.42x10^{9}	-7.88x10^{5}	10	7.6 %
$u(NC_{\text{NP RM}})$	1.47x10^{11}	1.40x10^{10}	g^{-1}	1.54x10^{-4}	2.16x10^{6}	60	56.8 %
$u(DF_{\text{NP RM}})$	6.22x10^{6}	8.40x10^{3}	--	-3.64	-3.06x10^{4}	60	0.0 %
$u(DF_{\text{Samp}})$	8.37x10^{2}	1.13	--	2.71x10^{4}	3.06x10^{4}	60	0.0 %
u(Rep)	1.000	0.057	--	2.27x10^{7}	1.29x10^{6}	3	20.3 %
**Combined Type A**					**1.85x10^{6}**		
**Combined Type B**					**2.18x10^{6}**		
**Combined standard uncertainty (u_c)**					**2.87x10^{6}**		

Table S-9. Individual Uncertainty Components for the spICP-MS Determination of Number Concentration of BCR-166 PS MP.

Component	Typical Value (x_i)	$u(x_i)$	units	c_i	c_iu_i	ν	Rel.
$u(n_{\text{NP events Samp}})$	745	23	particles	1.69x10^{4}	3.87x10^{5}	3	6.7 %
$u(t_{\text{analysis NP RM}})$	5.00	0.05	min	2.51x10^{6}	1.26x10^{5}	60	0.7 %
$u(q_{\text{liq NP RM}})$	0.015	5.6x10^{-4}	g min^{-1}	8.14x10^{8}	4.52x10^{5}	10	9.2 %
$u(n_{\text{NP events NP RM}})$	877	4.3	particles	-1.43x10^{4}	-6.18x10^{4}	2	0.2 %
$u(t_{\text{analysis Samp}})$	5.00	0.05	min	-2.49 x10^{6}	-1.24x10^{5}	60	0.7 %
$u(q_{\text{liq Samp}})$	0.015	5.6x10^{-4}	g min^{-1}	-7.86x10^{8}	-4.36x10^{5}	10	8.5 %
$u(NC_{\text{NP RM}})$	1.47x10^{11}	1.40x10^{10}	g^{-1}	8.55x10^{-5}	1.20x10^{6}	60	64.2 %
$u(DF_{\text{NP RM}})$	6.22x10^{6}	8.40x10^{3}	--	-2.02	-1.69x10^{4}	60	0.0 %
$u(DF_{\text{Samp}})$	6.25x10^{2}	8.4x10^{-1}	--	2.01x10^{4}	1.70x10^{4}	60	0.0 %
u(Rep)	1.000	0.037	--	1.26x10^{7}	4.66x10^{5}	3	9.7 %
**Combined Type A**					**8.75x10^{5}**		
**Combined Type B**					**1.21x10^{6}**		
**Combined standard uncertainty (u_c)**					**1.49x10^{6}**		

FIGURES

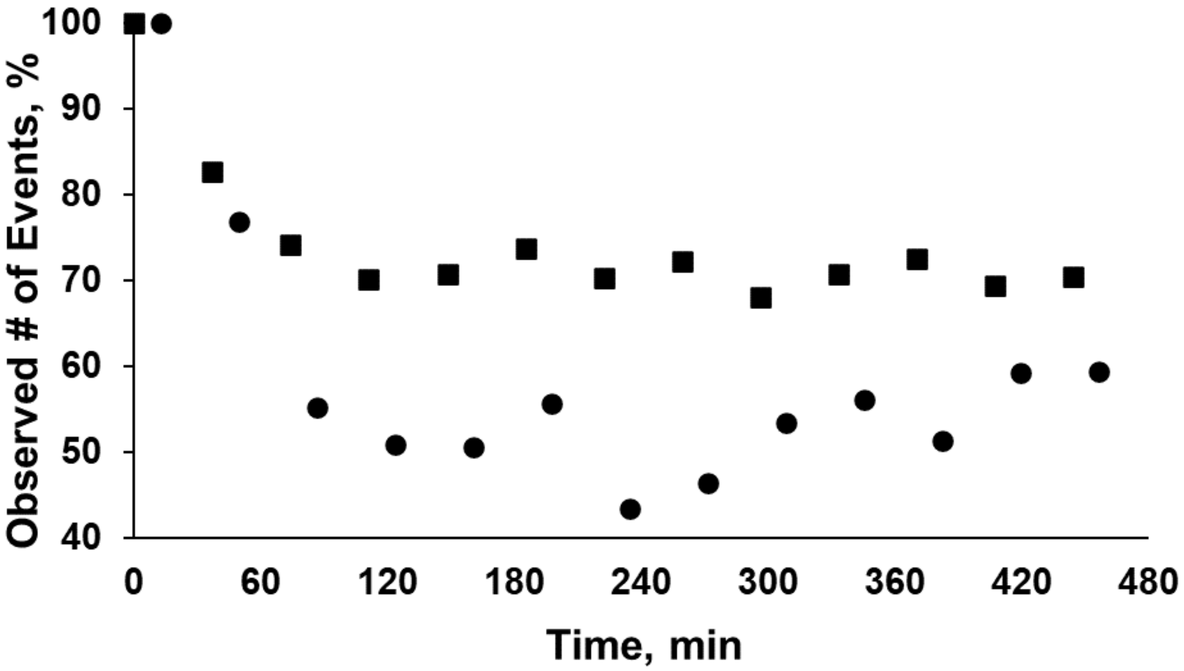

Figure S-1. Percentage of total events observed from the time of completion of sample preparation for samples of BCR-165 (■) and BCR-166 (●). Each data point represents a single analysis from n = 1 subsamples of each material. Samples were left undisturbed over 8 h.

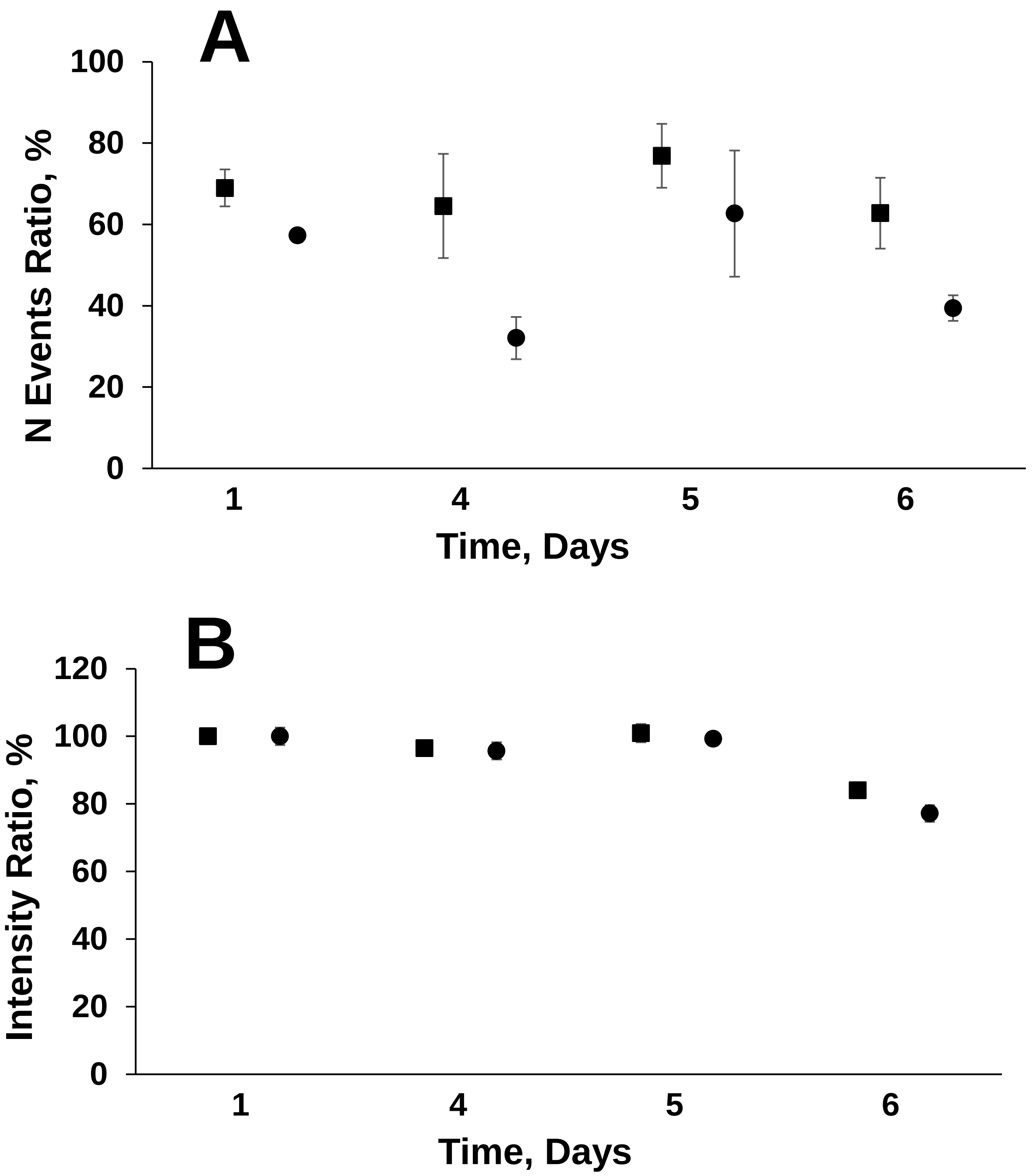

Figure S-2. Ratio of measured events in old samples vs. freshly prepared suspensions (A) and particle intensity ratio of old samples vs. freshly prepared suspensions (B) after one day (8 h), 4 d, 5 d, and 6 d for three replicates of n = 1 samples of BCR-165 (■) and BCR-166 (●). Error is represented by the standard deviation of measured ratios from three replicates.

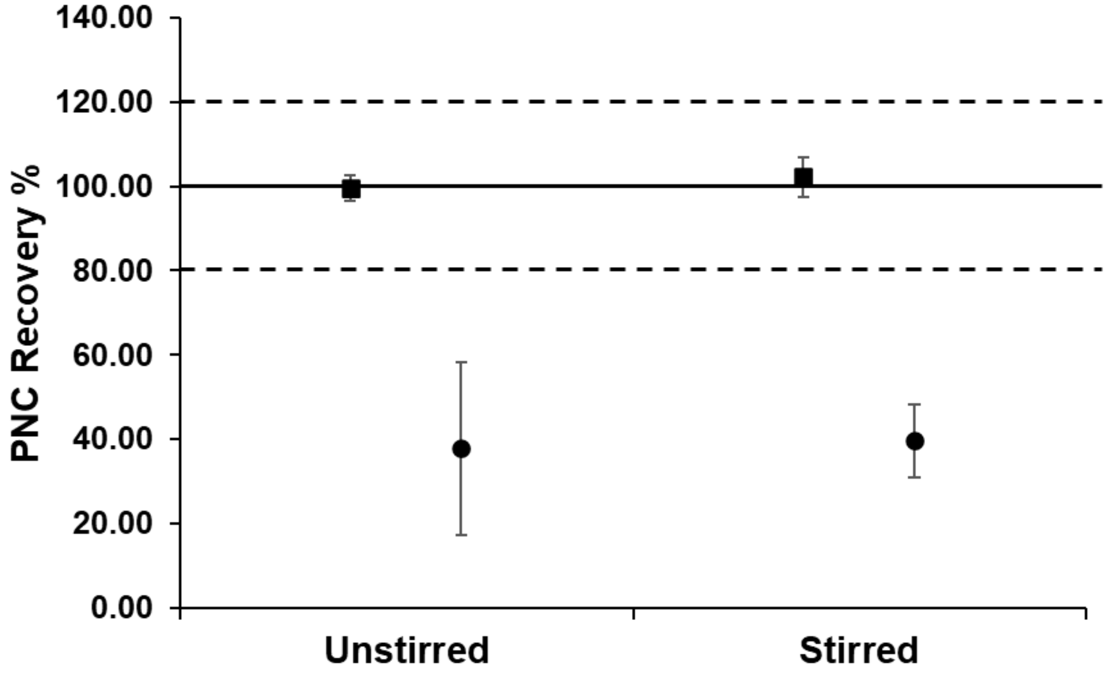

Figure S-3. Particle recovery percent of BCR-165 (■) and BCR-166 (●) particle suspensions analyzed at the standard nebulizer gas flow undisturbed and with active stirring for the analysis period. Measurements were conducted using three replicates of n = 1 samples for each particle suspension. Error is represented by the standard deviation of measured recoveries from the three replicate measurements.

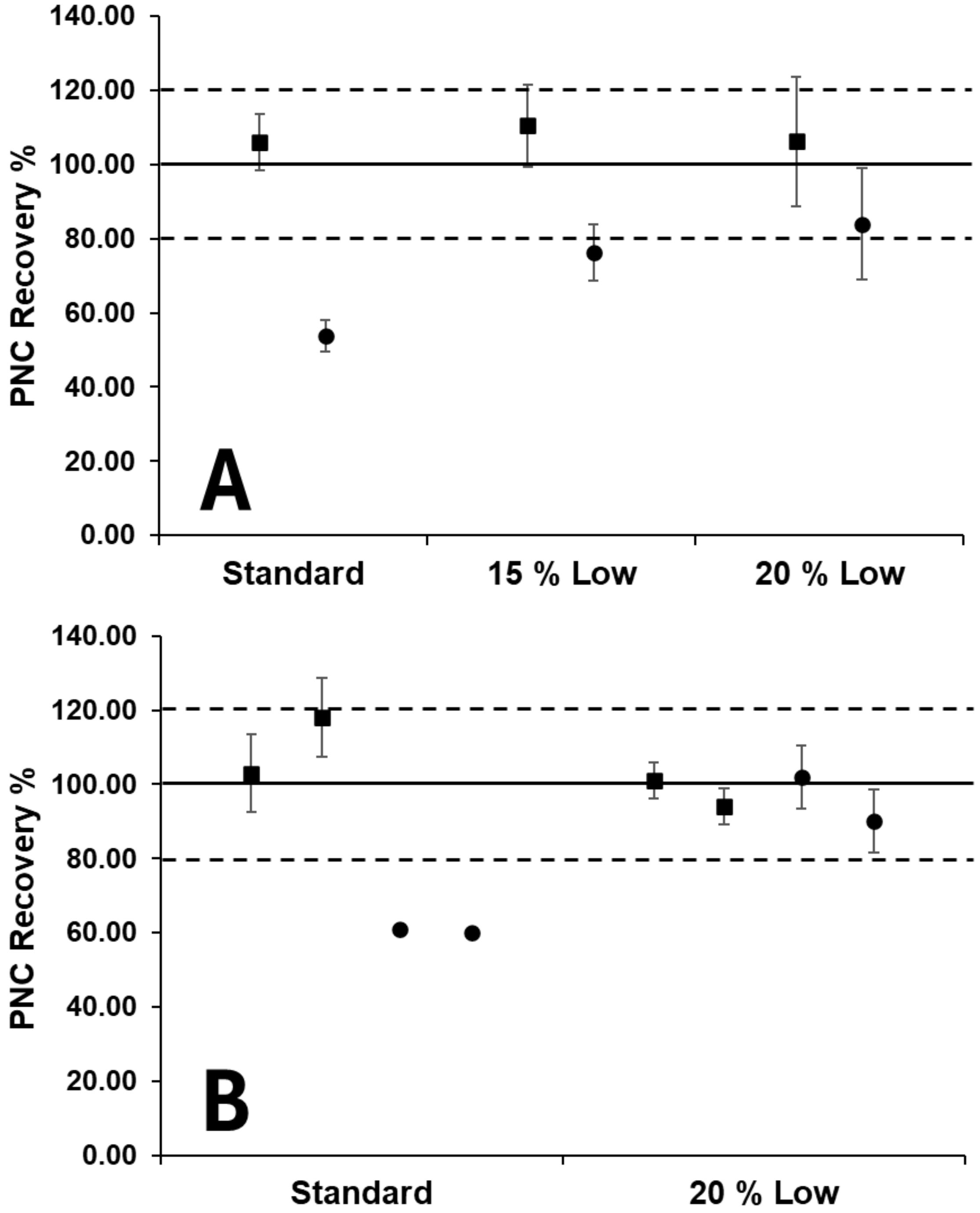

Figure S-4. Particle recovery percent of different nebulizer gas flow conditions tested during optimization (A) and repeatability testing of the optimum 20 % lowered nebulizer gas flow (B). Dilute particle suspensions of BCR-165 (■) and BCR-166 (●) were analyzed for one replicate each of n = 2 samples for each particle suspension. Error is represented by the standard deviation of measured recoveries from n = 2 measurements

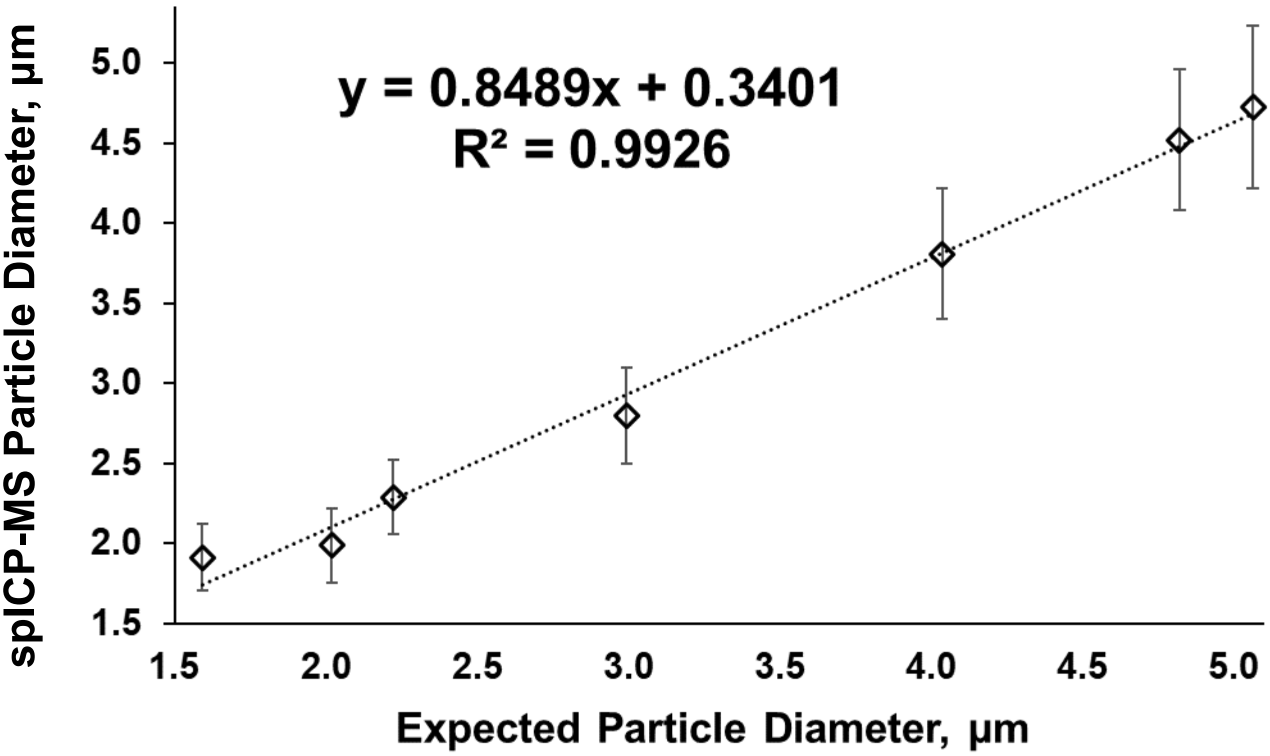

Figure S-5. Measured particle diameter vs. expected particle diameter of PS MPs with nominal sizes of 1.6 µm, 2.0 µm, 2.2 µm, 3.0 µm, 4.0 µm, 4.8 µm, and 5.0 µm measured using a reduced nebulizer gas flow (0.288 L min^{-1}) for n = 3 subsamples for each PS MP. Error represents the *U*95% C.I. of the spICP-MS measured particle diameter.

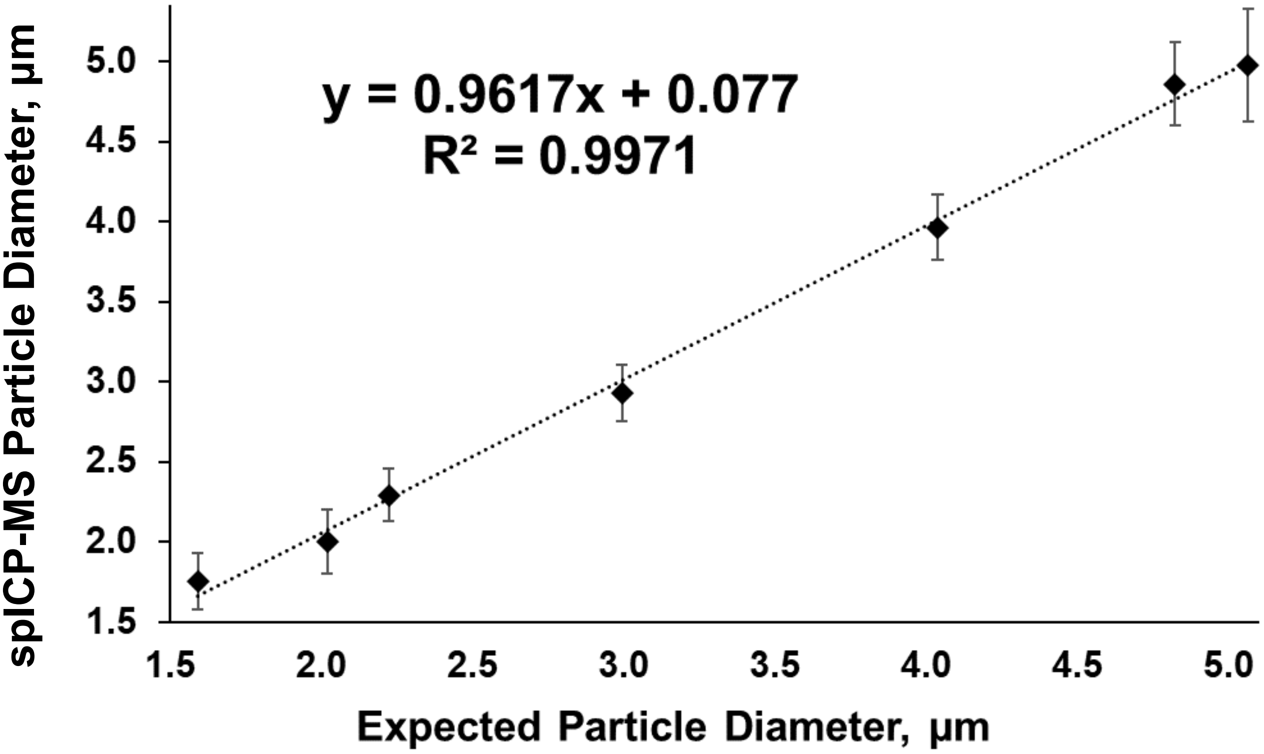

Figure S-6. Measured particle diameter vs. expected particle diameter of PS MPs with nominal sizes of 1.6 µm, 2.0 µm, 2.2 µm, 3.0 µm, 4.0 µm, 4.8 µm, and 5.0 µm measured using the standard nebulizer gas flow (0.36 L min^{-1}) for n = 3 subsamples for each PS MP. Error represents the *U*95% C.I. of the spICP-MS measured particle diameter.

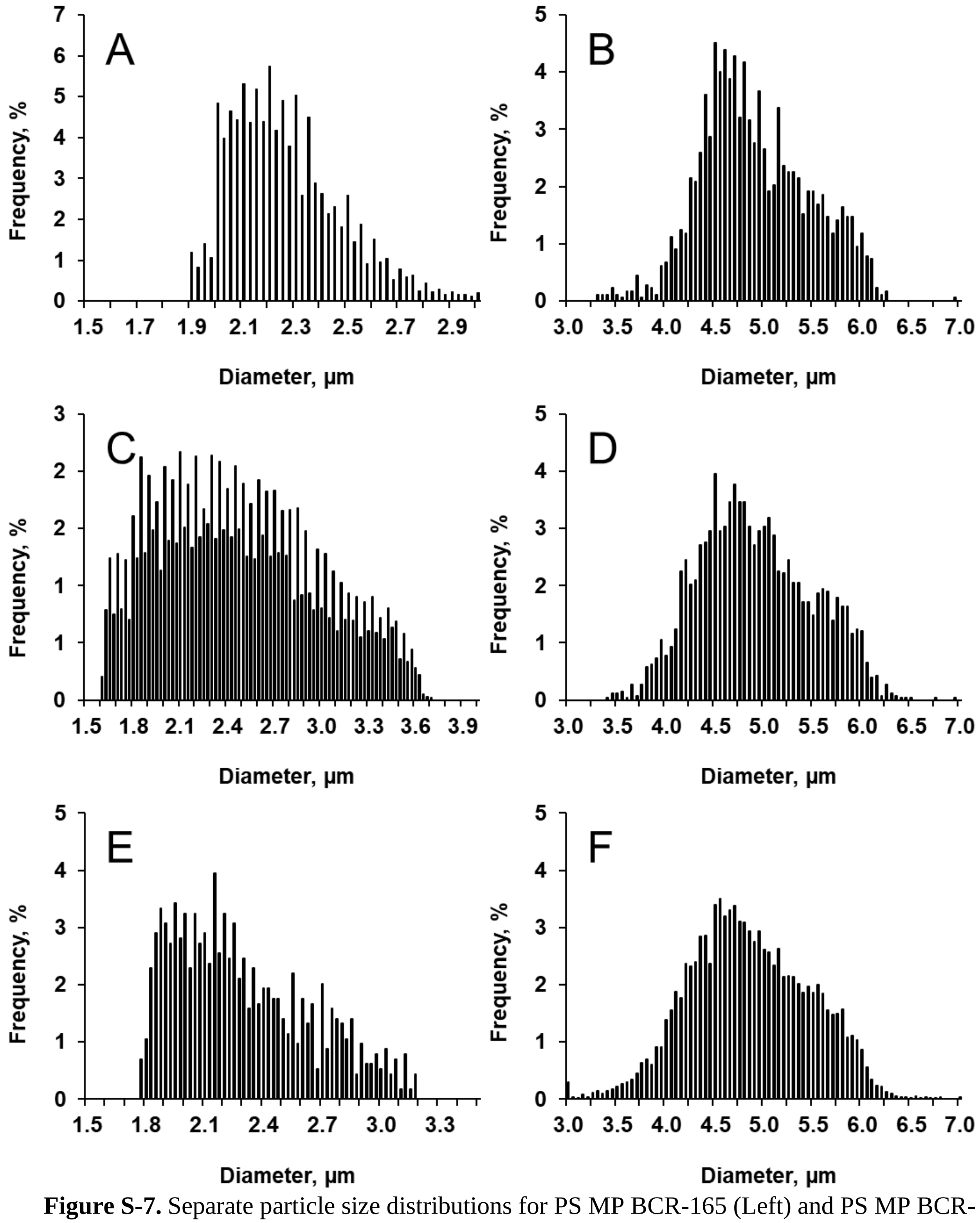

Figure S-7. Separate particle size distributions for PS MP BCR-165 (Left) and PS MP BCR-166 (Right) of analyzed mixtures with PNC ratios of 1:1 (A and B), 5:1 (C and D), and 1:5 (E and F) using the standard nebulizer gas flow of 0.36 L min^{-1}.

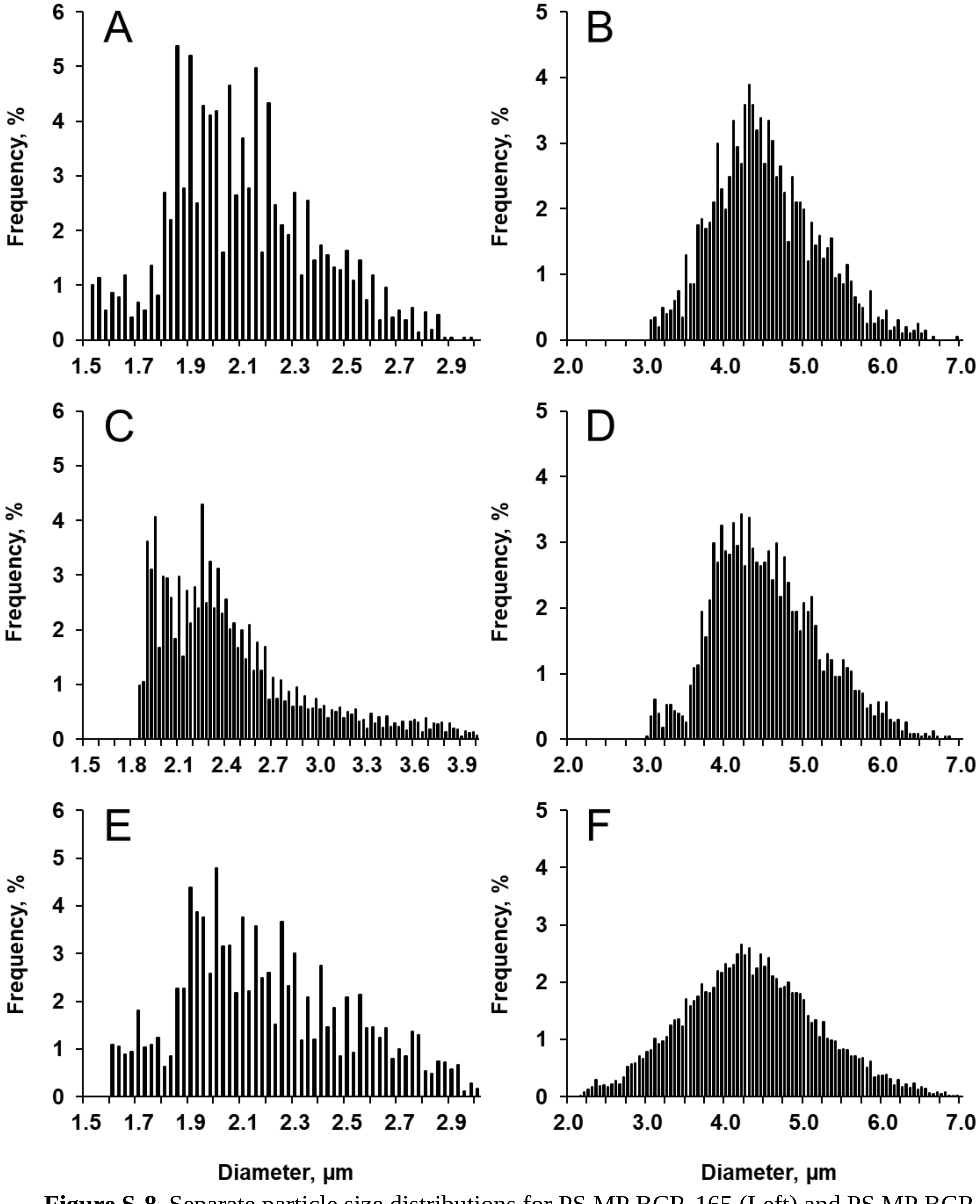

Figure S-8. Separate particle size distributions for PS MP BCR-165 (Left) and PS MP BCR-166 (Right) of analyzed mixtures with PNC ratios of 1:1 (A and B), 5:1 (C and D), and 1:5 (E and F) using the 20 % lower nebulizer gas flow of 0.288 L min^{-1}.

REFERENCES

(1) LGC. *Colloidal gold NPs – nominal diameter 30 nm Quality Control Material LGCQC5050, LGC*; 2019. https://www.lgcstandards.com/medias/sys_master/root/h84/h04/10435846012958/LGCQC5050.pdf.

(2) Murphy, K. E., Liu, J., Montoro Bustos, A.R., Johnson, M.E., Winchester, M.R. . Special Publication (NIST SP) - 1200-21, 2016. **2015**.

(3) Vetter, T. W. Quantifying measurement uncertainty in analytical chemistry–A simplified practical approach. In *Measurement Science Conference*, 2001.

(4) Tadjiki, S.; Montaño, M. D.; Assemi, S.; Barber, A.; Ranville, J.; Beckett, R. Measurement of the Density of Engineered Silver Nanoparticles Using Centrifugal FFF-TEM and Single Particle ICP-MS. *Analytical Chemistry* **2017**, *89* (11), 6056-6064. DOI: 10.1021/acs.analchem.7b00652.

(5) Garcia-Diez, R.; Sikora, A.; Gollwitzer, C.; Minelli, C.; Krumrey, M. Simultaneous size and density determination of polymeric colloids by continuous contrast variation in small angle X-ray scattering. *European Polymer Journal* **2016**, *81*, 641-649. DOI: https://doi.org/10.1016/j.eurpolymj.2016.01.012.

(6) Folzer, E.; Khan, T. A.; Schmidt, R.; Finkler, C.; Huwyler, J.; Mahler, H.-C.; Koulov, A. V. Determination of the Density of Protein Particles Using a Suspended Microchannel Resonator. *Journal of Pharmaceutical Sciences* **2015**, *104* (12), 4034-4040. DOI: https://doi.org/10.1002/jps.24635.

(7) Giddings, J. C.; Ho, J. Accurate Measurement of Density of Colloidal Latex Particles by Sedimentation Field-Flow Fractionation. *Langmuir* **1995**, *11* (7), 2399-2404. DOI: 10.1021/la00007a014.

(8) Pugh, T. L.; Heller, W. Density of polystyrene and polyvinyltoluene latex particles. *Journal of Colloid Science* **1957**, *12* (2), 173-180. DOI: https://doi.org/10.1016/0095-8522(57)90004-1.

(9) Sharp, D. G.; Beard, J. W. Size and density of polystyrene particles measured by ultracentrifugation. *Journal of Biological Chemistry* **1950**, *185* (1), 247-253. DOI: https://doi.org/10.1016/S0021-9258(18)56412-7.
